# Antibodies Against SARS-CoV-2 Nucleocapsid Protein Possess Autoimmune Properties

**DOI:** 10.3390/antib15010002

**Published:** 2025-12-22

**Authors:** Alexandra Rak, Yana Zabrodskaya, Pei-Fong Wong, Irina Isakova-Sivak

**Affiliations:** 1 Depatment of Virology and Immunology, Institute of Experimental Medicine, 197022 Saint Petersburg, Russia; po333222@gmail.com (P.-F.W.); isakova.sivak@iemspb.ru (I.I.-S.); 2 Department of Molecular Biology of Viruses, Smorodintsev Research Institute of Influenza, 197022 Saint Petersburg, Russia; zabryaka@yandex.ru

**Keywords:** autoimmunity, self-antigen, COVID-19, SARS-CoV-2, nucleocapsid protein, antiviral antibodies

## Abstract

Background/Objectives: Notwithstanding the declaration by the World Health Organization in May 2023 regarding the conclusion of the COVID-19 pandemic, new cases of this potentially lethal infection continue to be documented globally, exerting a sustained influence on the worldwide economy and social structures. Contemporary SARS-CoV-2 variants, while associated with a reduced propensity for severe acute pathology, retain the capacity to induce long-term post-COVID syndrome, including in ambulatory patient populations. This clinical phenomenon may be attributable to potential autoimmune reactions hypothetically triggered by antiviral antibodies, thereby underscoring the need for developing novel, universal vaccines against COVID-19. The nucleocapsid protein (N), being one of its most conserved and highly immunogenic components of SARS-CoV-2, presents a promising target for such investigative efforts. However, the protective role of anti-N antibodies, generated during natural infection or through immunization with N-based vaccines, alongside the potential adverse effects associated with their production, remains to be fully elucidated. In the present study, we aim to identify potential sites of homology in structures or sequences between the SARS-CoV-2 N protein and human antigens detected using hyperimmune sera against N protein obtained from mice, rabbits, and hamsters. Methods: We employed Western blot analysis of lysates from human cell lines (MCF7, HEK293T, THP-1, CaCo2, Hep2, T98G, A549) coupled with mass spectrometric identification to assess the cross-reactivity of polyclonal and monoclonal antibodies generated against recombinant SARS-CoV-2 N protein with human self-antigens. Results: We showed that anti-N antibodies developed in mice and rabbits exhibit pronounced immunoreactivity towards specific components of the human proteome. In contrast, anti-N immunoglobulins from hamsters showed no non-specific cross-reactivity with either hamster or human proteomic extracts because of the lack of autoreactivity or immunogenicity differences. Subsequent mass spectrometric analysis of the immunoreactive bands identified principal autoantigenic targets, which were predominantly heat shock proteins (including HSP90-beta, HSP70, mitochondrial HSP60, and HSPA8), histones (H2B, H3.1–3), and key metabolic enzymes (G6PD, GP3, PKM, members of the 1st family of aldo-keto reductases). Conclusions: The results obtained herein highlight the differences in the development of anti-N humoral responses in humans and in the Syrian hamster model. These data provide a foundational basis for formulating clinical recommendations to predict possible autoimmune consequences in COVID-19 convalescents and are of critical importance for the rational design of future N protein-based, cross-protective vaccine candidates against novel coronavirus infections.

## 1. Introduction

The pandemic of the novel coronavirus disease COVID-19, the causative agent of which is SARS-CoV-2, has significantly affected the state of the global economy and social sphere. SARS-CoV-2 continues to circulate in the human population, and new antigenic variants have emerged [1]. The symptoms of the infection they cause are generally mild compared to the consequences of infection with the ancestral B.1 variant [2], but the risk of developing long-term post-COVID syndrome still remains [3,4]. One of the key factors contributing to this phenomenon may be the adverse effects of antiviral antibody production, including autoimmune reactions and antibody-dependent enhancement of infection (ADE).

Consequently, the development of novel cross-protective vaccines against COVID-19 remains a pertinent scientific objective. The nucleocapsid (N) protein emerges as a highly promising candidate antigen due to its pronounced immunogenicity and significant evolutionary conservation, which exceeds that of the spike protein traditionally targeted by diagnostics and vaccines [5]. According to previous studies, the few substitutions that arise in the N protein sequence during SARS-CoV-2 evolution [6] are localized mainly in the N-terminal region, RNA-binding domain, and intermediate linker. These mutations can impact immunogenic T- and B-cell epitopes, thereby reflecting an evolutionary strategy for immune evasion [7]. The relative conservatism of the N protein permits the utilization of the ancestral B.1 strain’s protein as a screening antigen for detecting infections caused by recent SARS-CoV-2 variants [8,9,10]. Nonetheless, a study in a murine model demonstrated differences in the immunogenicity and response patterns induced by recombinant N proteins from different lineages, underscoring the functional impact of evolutionary changes on N antigenicity [11].

As an internal viral component, the N antigen is conventionally recognized for its role in inducing robust T-cell-mediated immunity. However, it also elicits a substantial humoral response, characterized by the active production of anti-N antibodies following both natural infection and vaccination [12]. The high immunogenicity of the N protein is likely attributable to its abundant synthesis within the cytoplasm of infected cells and its demonstrated presence on the cell surface, which nevertheless remains a discussion subject [13,14,15]. The magnitude and persistence of the anti-N antibody response in COVID-19 convalescents—lasting up to eighteen months post-infection [8,16] are comparable to those of antibodies targeting exposed antigens such as the spike protein and its receptor-binding domain (RBD) [17].

The mechanisms by which anti-N antibodies contribute to antiviral protection remain largely undefined. Although they lack direct virus-neutralizing activity in vitro, these antibodies are capable of mediating effector functions of the innate immune system, including the initiation of the complement cascade [18] and antibody-dependent cellular cytotoxicity/phagocytosis (ADCC/ADCP) [19]. Furthermore, serum raised against the N protein of the B.1 variant has been shown to confer protection to Syrian hamsters against challenge with a homologous virus [20]. Given these findings, vaccines designed to stimulate anti-N antibodies would appear to be an ideal strategy for combating COVID-19. This prospect, however, is tempered by recent reports indicating that anti-N antibodies can exhibit cross-reactivity with human self-antigens [21] and potentially exacerbate disease severity, thereby posing a risk for triggering autoimmune pathologies and ADE of infection [22,23].

In this study aimed to detect human-derived proteins homological to N antigen and to assess undesirable effects of the generation of anti-N antibodies, we evaluated the autoreactive potential of a panel of previously characterized murine monoclonal antibodies against the N protein of the B.1 variant, alongside hyperimmune sera from rabbit, mouse, and hamster models. The reactivity of these immunoglobulins was assessed against the constitutive proteome of various human-derived cell lines and the proteome of the Syrian hamster (represented by the CHO cell line). Immunoreactive bands indicative of autoreactivity were identified and subsequently characterized by mass spectrometry. A comprehensive bioinformatics analysis was then employed to identify potential regions of structural or sequential homology between the SARS-CoV-2 N protein and the identified human self-antigens.

## 2. Materials and Methods

### 2.1. Cells, Sera, and Protein

The cell cultures of cell lines MCF7, HEK293T, THP-1, CaCo2, Hep2, T98G, A549, and CHO were purchased from the American Type Culture Collection (ATCC, Manassas, VA, USA) and maintained in DMEM (except for RPMI for THP-1) supplemented with 10% fetal bovine serum (FBS) and 1× antibiotic–antimycotic (AA) (all from Capricorn Scientific, Ebsdorfergrund, Germany). These particular cell lines were chosen due to their human origin and multiple tissue derivation, primarily epithelial, which is important to analyze as a primary cell type for viral entry.

For this study, hyperimmune sera against recombinant N(B.1) protein were generated in five female BALB/c mice (aged 6–8 weeks, 16–18 g), three male rabbits (aged 6–7 months, 3–3.5 kg), and five female Syrian hamsters (aged 4–8 weeks, 80–100 g) as standard model animals appropriate for virological and serological studies for subsequent use in Western blot assay. For this, N(B.1) antigen was expressed in a bacterial system (*E. coli* BL21(DE3) cells) and an IMAC-purified system using a previously developed protocol [8].

The use of animals to obtain hyperimmune sera was approved by the Ethics Committee of the Institute of Experimental Medicine (protocol No. 4/24, dated 24 October 2024). The alternative options (e.g., in vitro B cell stimulation, immunized splenocytes) were not applicable as sera with high anti-N titers were needed to conduct the study. The number of animals used was the minimum statistically acceptable number, and complied with the “Three Rs” recommendations; no additional sample size calculations were performed. The animals were housed in a standard laboratory vivarium facility in the standard plastic cages and sawdust bedding with free access to food and water under standard light-dark cycle conditions and at room temperature. The quarantine period before the study was 2 weeks. The animals were randomly assigned to groups, and the final experimental product was the individual serum samples, which were further pooled. The qualified personnel were aware of the group assignment, immunizations, sera collection and analysis at all stages of the study.

Naïve animals with certified health status (purchased from Stezar commercial breeder (Krasnogvardeisky village, Vladimir region, Russia)) were pre-screened in ELISA on 2 µg/mL immobilized recombinant N(B.1) protein; no pre-existing reactivity was detected. Animals were immunized thrice intraperitoneally/intraperitoneally/subcutaneously with 10/100/500 µg (for mice/hamsters/rabbits, respectively) of recombinant N(B.1) protein emulsified with aluminum hydroxide (1:3), with a 14-day interval under a mild ether anesthesia as a safe, fast, non-invasive, and available analgesia method. All the immunizations were carried out by qualified personnel in a strict order to avoid possible confounders. Immunized animals were monitored daily, and sera was collected humanely at 14 days after the last injection under the mild ether anesthesia. The resulting titer of pooled anti-N(B.1) sera was assessed in solid-phase ELISA on immobilized N(B.1) protein. The titers of naïve animal sera (used as negative control) were also checked side-by-side and appeared to be lower than the ELISA sensitivity threshold.

The study also used mouse hyperimmune serum against the recombinant NP protein of the influenza virus strain A/Leningrad/134/17/57 (H2N2), obtained in a manner similar to that described above.

Previously generated hybridoma-derived mouse monoclonal antibodies NCL2 and NCL10 [11] were also used to reveal the zones of non-specific reactivity and as a positive WB control. These antibodies were generated to N(B1) protein, but they are able to universally recognize N proteins of evolutionary distant SARS-CoV-2 lineages.

Anti-IFNα mouse monoclonal antibody ab191903 (Abcam, Cambridge, UK) was used as a negative WB control.

### 2.2. SDS-PAGE and Western Blot

The sodium dodecyl sulfate–polyacrylamide gel electrophoresis (SDS-PAGE) in reducing conditions was performed by a standard method [24] and was used to separate protein components of cell lysates prior to the development with anti-N antibodies by Western blotting with subsequent mass spectrometry identification. For each cell line, lysis of 10^6^ cells in PBS was performed on ice using ultrasonic pulse sonication (three rounds of 30 s each with a 20-s break) at an amplitude of 30%. Laemmli loading buffer containing beta-mercaptoethanol (at final concentration 1%) was added to the obtained lysates followed by sample heating at 95 °C for 5 min. The protein load per gel lane was ~5 μg, and each sample was analyzed in triplicates. Proteomes were resolved on a 10% polyacrylamide gel at 120 V for 1 h before being stained with colloidal Coomassie G-250 solution (Bio-Rad, Hercules, CA, USA) for 1 h at room temperature or semi-dry transferred to 0.45 μm nitrocellulose membranes for 2 h at 100 V. Blots were blocked overnight at 4 °C with 5% skimmed milk in PBS-T and then treated with anti-N mAbs (5 μg/mL) or anti-N(B.1)/anti-NP(H2N2) sera (diluted 1:100) in blocking buffer for 1 h at 37 °C. Then, species-specific HRP-conjugated secondary antibodies (Bio-Rad, Hercules, CA, USA) diluted 1:3000 in blocking solution were added to the triple-washed blots for 1 h at 37 °C. After three washes with PBS-T, the blots were developed with 0.05% solution of diaminobenzidine (Sigma, St. Louis, MO, USA) in PBS containing 1% hydrogen peroxide. Finally, the membranes were washed with water and the images were captured using Gel Doc EZ Gel Documentation System (Bio-Rad, Hercules, CA, USA).

### 2.3. MALDI-TOF Mass-Spectrometry

Mass spectrometry identification of proteins was performed according to the previously proposed MALDI-TOF protocol [25]. To analyze the amino acid composition of proteins, enzymatic hydrolysis in gel by trypsin was performed after SDS-PAGE. For this, gel fragments containing the areas of interest were excised (∼1 cm^3^), destained from the Coomassie G-250 (twice in 150 μL each of 30 mM ammonium bicarbonate solution and 40% acetonitrile in water), dehydrated in 100% acetonitrile, and air-dried. Then, 2 μL of trypsin solution (20 μg/mL in 50 mM ammonium bicarbonate) was added to the gel fragments and incubated at 37 °C for 18 h. The reaction was stopped with 3 μL of 1% TFA, 10% acetonitrile in water. For protein identification, the resulting set of tryptic peptides was mixed with DHB matrix (Bruker, Bremen, Germany) in equal volumes, applied to a steel target, and examined in reflectance positive ion detection mode on an UltrafleXtreme MALDI-TOF/TOF mass spectrometer (Bruker, Bremen, Germany). At least 5000 laser pulses were summed for each spectrum. For spectra calibration internal standards (ions, corresponding to trypsin autoproteolysis) were used. Protein identification was performed using MASCOT search engine [26] by accessing the UniProt database (taxonomy—*Homo sapiens*) [27]. Methionine oxidation and deamidation were indicated as variable modifications. The accuracy of mass determination was limited to 20 ppm. Up to one trypsin error (omission of a proteolysis site) were allowed.

### 2.4. Protein Set and Structural Templates

Given the absence of previously published X-ray crystallographic structures for the full-length nucleocapsid (N) protein, the two primary structured domains of the SARS-CoV-2 N protein—the N-terminal RNA-binding domain (NTD) and the C-terminal dimerization domain (CTD)—were analyzed independently. The structural references for this analysis were Protein Data Bank (PDB) entries 8IQJ (NTD) and 8W6W (CTD). For each candidate human self-antigen, a high-resolution structure from the PDB was utilized (as specified in the Supplementary Data). In cases where only an amino acid sequence was available, the corresponding PDB structure of the closest human ortholog or homologous domain was employed for alignment.

All structural alignments were conducted using PyMOL v3.1.6.1. For each human protein target, both N-domains (NTD and CTD) were independently compared using two distinct algorithms: (1) the Align method, which executes a global sequence alignment followed by a structural fit with iterative outlier rejection, and (2) the Super method, which performs a sequence-independent, dynamic programming-based structural alignment with subsequent refinement. The resulting alignment scores from both the Align and Super functions in PyMOL were recorded for each protein-domain pair.

As the raw Align and Super scores obtained from PyMOL are inherently variable across different protein structures and domains, precluding direct comparability, we normalized all values within each domain (N-CTD and N-NTD) separately using min–max scaling to allow consistent ranking:

$${\mathrm{NormScore}}_{i,d}=\frac{S_{i,d}-S_{d}^{min}}{S_{d}^{max}-S_{d}^{min}}$$

where $S_{i,d}$ is the raw score (Align or Super) for protein *i* in domain *d*, and $S_{d}^{min}$ and $S_{d}^{max}$ are the minimum and maximum values across all proteins for that domain.

This procedure rescales the distribution of scores in each domain to the range [0,1], with: 0 = weakest similarity in that domain, 1 = strongest similarity in that domain.

For each protein, a combined normalized score was then defined as follows:

$${\mathrm{Combined}\;\mathrm{NormScore}}_{i}=\max\left({\mathrm{NormScore}}_{i,CTD},\;\;{\mathrm{NormScore}}_{i,NTD}\right)$$

The driver domain was defined as the domain (CTD or NTD) that contributed the higher normalized value for a given protein.

## 3. Results

### 3.1. Autoimmune Properties of Anti-N Antibodies

**Figure 1:**
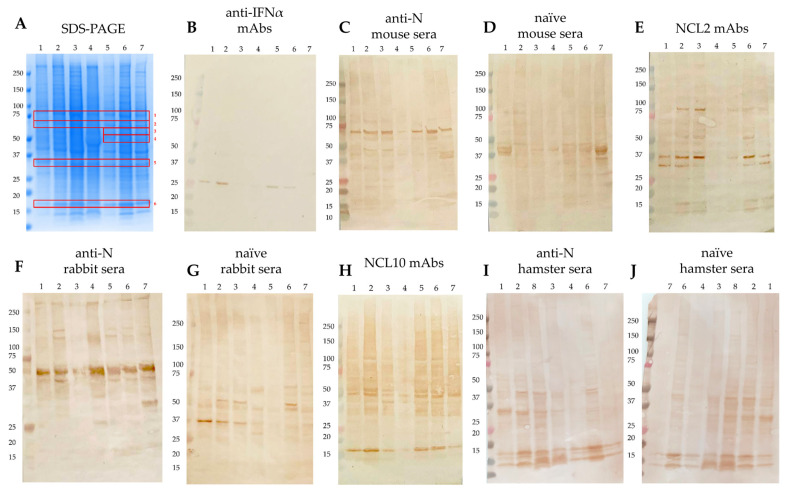
SDS-PAGE demonstrating autoreactivity zones (**A**) and Western blot analysis of cell lyzates developed with different antibodies: anti-IFNα mAbs (**B**), anti-N mouse sera (**C**), naïve mouse sera (**D**), NCL2 mAbs (**E**), anti-N rabbit sera (**F**), naïve rabbit sera (**G**), NCL10 mAbs (**H**), anti-N hamster sera (**I**), naïve hamster sera (**J**). The revealed bands are framed in red, and the red numbers indicate the mass spectrometry identification zones. The following cell lines are designated by the numbers: 1—MCF7; 2—HEK293; 3—THP1; 4—CaCo-2; 5—Hep2; 6—T98G; 7—A549; 8—CHO.

Western blot analysis of the human-derived cell lyzates demonstrated substantial cross-reactivity between anti-N (B.1) monoclonal antibodies, as well as sera from immunized animals, and multiple components of human proteome (Figure 1). Sera from corresponding naïve animals and anti-IFNα monoclonal antibody served as negative controls and showed no such reactivity. Notably, positive bands were consistently observed across all cell lysates examined, with an identical banding pattern, indicating a lineage-independent recognition of human proteins by the anti-N antibodies. Of particular interest, the reactivity of anti-N (B.1) antibodies to the human antigens was demonstrated for A549 (derived from lung adenocarcinoma), Hep2 (larynx carcinoma), and Caco2 (colorectal adenocarcinoma) eukaryotic cell lines. Given that epithelial tissues represent a primary target for SARS-CoV-2 infection, this finding suggests a potential link between the observed autoimmunity in vitro and viral tropism in vivo.

As a biological control, the same cell lysates were developed with mouse hyperimmune sera against the NP protein of the influenza A virus (H2N2). This antigen has been chosen as sharing similar structural features with N protein as an internal viral component. In addition, anti-NP antibodies are known to be actively induced upon influenza infection and immunization with live attenuated or inactivated whole virion influenza vaccines [28,29], with no safety signals of developing autoimmune consequences after infection and vaccination.

**Figure 2:**
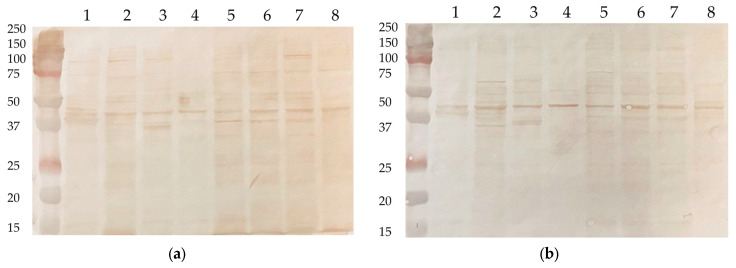
Western blot analysis of cell lysates developed with anti-NP mouse sera (**a**) or naïve mouse sera (**b**). 1—MCF7; 2—HEK293; 3—THP1; 4—CaCo-2; 5—Hep2; 6—T98G; 7—A549; 8—CHO.

No zones of reactivity of anti-NP antibodies with human proteins were detected (Figure 2), indicating the unique properties of anti-N(B.1) antibodies, as well as the absence of a risk of provoking autoimmune pathologies by anti-NP(H2N2) antibodies and the feasibility of stimulating the production of these IgGs with NP-bearing influenza vaccines.

### 3.2. Mass Spectrometry Identification of Bands Developed with Anti-N Antibodies

Then, a composition of the major revealed bands was identified by MALDI-TOF mass-spectrometry analysis (Table 1). The identification was performed for proteins contained in each single detected band.

As a result of mass spectrometric analysis of the detected bands, the main possible intrinsic target antigens of autoreactive anti-N antibodies were identified. Among them were mainly heat shock proteins (HSP90-beta, HSP70, mitochondrial HSP60, HSPA8), histones (H2B, H3.1—3), and metabolic enzymes (G6PD, GP3, PKM, aldo-ketoreductase-1 family proteins).

A detailed sequence/structure alignment of the N protein and human antigens binding to anti-N antibodies is presented in Table S2.

### 3.3. Interpretation of Normalized Align vs. Super Scores

Then, we compared the similarity of the amino acid sequences of N (B.1) with the primary structures of the detected proteins contained in the analyzed lysates and identified by mass spectrometry. Analysis of the amino acid composition and structure of the predicted autoantigens with those of the N protein SARS-CoV-2 showed the presence of common regions.

The Align result emphasized linear motif similarity, with high scores for histones (H3.1–3.3, H2B), HSP90α/β, PPIA, and an inflated outlier for PKM. This suggests that the N protein shares short (12—55), basic or helix-rich common motifs with these proteins of human origin (Table 2).

Super (sequence-independent structural fit) emphasized fold mimicry, highlighting CALR, IGHG1, HSP70/90, aldo-keto reductases (AKR family), PKM, and CH60. These hits revealed conformational overlap between N-NTD/CTD and abundant human proteins involved in stress responses, metabolism and immunity (Table 3).

Consensus across both methods points to HSP70/90, PKM, and histones as robust cross-reactive candidates, in line with Western blot/MALDI-TOF identifications. Together, the dual analysis supports the idea that anti-N antibodies may recognize human proteins via both sequence motifs and structural folds, providing a mechanistic basis for observed autoreactivity.

## 4. Discussion

Antibody-Dependent Enhancement (ADE) represents a complex and incompletely elucidated phenomenon periodically observed in viral infections, necessitating a re-evaluation of strategies that prioritize enhanced induction of the humoral mode of immunity [30]. The probability of ADE developing varies across different infections, remains largely unpredictable, and is usually determined empirically [31]. The cases of ADE development have been reliably shown to be a result of the use of vaccines against dengue and Zika fevers [32,33,34,35] and respiratory syncytial infection [36]. While some researchers believe that the development of ADE from COVID-19 vaccination is unlikely [37,38] or may only occur in rare cases [39,40], a prevailing consensus suggests this phenomenon may be a hallmark of coronavirus infections [40,41,42,43], and therefore, the occurrence of this pathological condition upon COVID-19 warrants serious concern [23,44]. In particular, the binding of antiviral antibodies to Fc receptors localized on macrophages or mast cells is considered to be the most probable mechanism for the ADE development [45,46], potentially triggering Multisystem Inflammatory Syndrome in Children (MIS-C), in Adults (MIS-A), or Long COVID syndrome [47,48,49].

FcγRIIA and FcγRIIIA receptors are considered to be principal mediators of IgG-initiated ADE [50]. Additionally, complement cascade activation by antiviral antibodies presents an alternative pathway for its development [51,52]. Thus, although anti-SARS-CoV-2 antibodies are considered to be a powerful tool for antiviral defense [46,53], an association between their serum levels and ADE risk has been demonstrated experimentally [38,54]. It is hypothesized that antibodies lacking neutralizing activity may contribute most significantly to ADE [22,23,55,56], with the associated risks appearing to be strain-independent [56,57]. In this regard, the use of synthetic aptamers against SARS-CoV-2 as an alternative to antibodies to avoid the development of ADE seems attractive [58,59]. At the same time, there is a hypothesis about an FcR-independent mechanism of ADE development in COVID-19 [23], supported by in vitro data showing that convalescent antibodies may not always induce ADE [60,61,62].

Undesirable effects of the formation of anti-N antibodies may be associated not just with the ADE development. The ability of monoclonal N-specific antibodies against SARS-CoV-2 [18] and anti-N antibodies from COVID-19 convalescents [63] to induce the initiation of the complement cascade has been previously demonstrated. This involvement of N-specific antibodies in innate immune effector functions necessitates careful study to assess the risk of autoimmune pathologies, particularly given their documented cross-reactivity with human autoantigens [21,64,65]. The similar ability of non-neutralizing COVID-19-associated antibodies to provoke pathogenic effects, such as hypercoagulable and proinflammatory states, has been described previously [66,67].

Other putative causes of the damaging action of anti-N antibodies, in addition to ADE, may be autoimmune phenomena such as epitope spreading, which involves broadening the immune responses from a single epitope to target additional ones [68], and molecular mimicry with host proteins, typical of viral antigens [69]. In addition, hypothetically, the detrimental effects of anti-N antibody generation may be unrelated to autoimmune reactions, and may be associated with the richness of N protein with the basic and disordered regions, which may explain the “sticky” nature of anti-N antibodies and may provoke non-specific binding.

In the present study, we conclusively demonstrate that anti-N antibodies elicited in mice and rabbits immunized with recombinant N protein bind human self-antigens. We subsequently identified the primary autoantigenic targets and regions of structural homology. Bioinformatic analysis revealed the greatest sequence similarity (via the Align algorithm) between the N-protein’s C-terminal domain (CTD) and heat shock protein HSP 90-beta, and between its N-terminal domain (NTD) and pyruvate kinase PKM (Table 2). Structural alignment (via the Super algorithm) indicated the highest similarity between the CTD and aldo-keto reductase family 1 member C3, and between the NTD and calreticulin (Table 3). Considering that the separation of protein components of lysates was carried out under reducing conditions linearizing molecular structures (Figure 1), the sequence-based similarities identified by the Align algorithm are most congruent with our experimental findings. These data are in line with the oligomeric nature of these proteins [70,71], which are also typical for nucleoprotein, and with the previously published reports on the ability of some viral antigens to molecularly mimic the host cell components [69]. Given the high expression levels of these identified autoantigens in neurological tissue [72,73], our findings suggest a potential mechanistic link to the neuropathological manifestations observed in post-COVID syndromes. However, this assumption is undoubtedly hypothetical and requires further confirmation in in vivo experiments.

Our study has several obvious limitations. First of all, in this study we used the one-dimensional discontinuous SDS-PAGE method, which does not allow for the precise separation of proteins with similar molecular masses. It should be also noted that the results may have been influenced by antibody “stickiness” under reducing conditions, although adequate experimental controls were used. We also did not perform functional analyses of autoreactivity, e.g., when uninfected human cell lysates are used as coating antigens in ADCC/CDC assays. However, using SARS-CoV-2 infected cells or recombinant N protein, we previously demonstrated the capacity of anti-N antibodies to induce CDC and ADCC activity [20], suggesting that anti-N antibodies binding to human autoantigens can also elicit similar functional activity. Furthermore, since this paper reports on the findings of preliminary experiments, we did not perform WB densitometry or investigate the ability of autoreactive anti-N antibodies of various origins to bind to the purified or recombinant human-derived proteins. Consequently, the results have not undergone validation. Moreover, in this qualitative study, the affinity and kinetics of binding of anti-N antibodies to human antigens were not investigated by ELISA or SPR methods. These limitations need to be addressed upon further research in this direction.

## 5. Conclusions

The demonstrated autoreactivity of anti-nucleocapsid antibodies against specific human proteomic components necessitates a cautious approach toward immunization strategies designed to elicit a robust anti-N humoral response. These findings provide a foundational basis for predicting autoimmune sequelae in COVID-19 convalescents and inform the rational design of next-generation N protein-based vaccines. The practical implications for such vaccine design include preferential stimulation of T-cell mediated immunity by vectored vaccines, induction of non-neutralizing antibodies, monitoring of possible ADE-like effects, and use of N protein not as the main, but as an additional vaccine component.

The paper does not confirm the role of antibodies generated against N protein in development of autoimmune pathologies in vivo, but only demonstrates the potential for autoreactivity of anti-N antibodies in vitro. Further investigation is required to precisely delineate the full spectrum of autoantigens targeted by these antibodies in vivo.

## Figures and Tables

**Table 1:** The proposed targets for autoreactivity of anti-N antibodies.

Band Number */ kDa	1 (MCF7)	2 (HEK293)	3 (THP-1)	4 (CaCo-2)	5 (Hep2)	6 (T98G)	7 (A549)
1 (85)	HS90B and/or HS90A	HS90B and/or HS90A	HS90B and/or HS90A	HS90B	HS90B and/or HS90A	HS90B and/or HS90A	HS90B and/or HS90A
2 (70)	HSP7C	HSP7C	HSP7C	HSP7C	HSP7C	HSP7C + HSP71	HSP7C + HSP71
3 (60)					KPYM	KPYM + CH60	KPYM
4 (50)					CAP1	CALR	G6PD
5 (30)	AK1BA + (AK1C1 and/or AK1C3) + G3P	G3P	G3P	G3P	G3P	G3P + LDHB	G3P
6 (17)	H31 or H32 or H33	PPIA + H3	PPIA	(H31 or H32 or H33) + PPIA + H2B	IGHG1	(H3 or H32 or H33) + H2B + PPIA	H3 + H2B

* the band number is given according to Figure 1A. HS90B_HUMAN—Heat shock protein HSP 90-beta; HS90A_HUMAN—Heat shock protein HSP 90-alpha; HSP7C_HUMAN—Heat shock cognate 71 kDa protein; HSP71_HUMAN—Heat shock 70 kDa protein 1A/1B; KPYM_HUMAN—Pyruvate kinase PKM; CH60_HUMAN—60 kDa heat shock protein, mitochondrial; CAP1_HUMAN—Adenylyl cyclase-associated protein 1; CALR_HUMAN—Calreticulin; G6PD_HUMAN—Glucose-6-phosphate 1-dehydrogenase; AK1BA_HUMAN—Aldo-keto reductase family 1 member B10; AK1C1_HUMAN—Aldo-keto reductase family 1 member C1; AK1C3_HUMAN—Aldo-keto reductase family 1 member C3; G3P_HUMAN—Glycer-aldehyde-3-phosphate dehydrogenase; LDHB_HUMAN—L-lactate dehydrogenase B chain; H31_HUMAN—Histone H3.1; H32_HUMAN—Histone H3.2; H33_HUMAN—Histone H3.3; PPIA_HUMAN—Peptidyl-prolyl cis-trans isomerase A; H2B_HUMAN—Histone H2B; IGHG1_HUMAN—Ig gamma-1 chain C region. All proteins were reliably identified (*p* < 0.05), as the Mascot Score exceeded the threshold. Detailed information on Mascot scoring is presented in Table S1.

**Table 2:** Scores calculated from alignments of NTD and CTD of N protein and sequences of the detected human cross-reactive antigens performed by Align algorithm. For each protein, a combined normalized score is defined as maximal normalized CTD or NTD score.

Target	Align CTD	Align NTD	Norm Align CTD	Norm Align NTD	Combined Norm Align	Driver Domain Align	Rank Align
HS90B	77	38.5	1	0.119	1	CTD	1
KPYM	51.5	178.5	0.49	1	1	NTD	1
HS90A	76.5	40.5	0.99	0.132	0.99	CTD	3
CALR	68	35	0.82	0.097	0.82	CTD	4
H32	66.5	61	0.79	0.261	0.79	CTD	5
HSP7C	57	61	0.6	0.261	0.6	CTD	6
H2B	54.5	55	0.55	0.223	0.55	CTD	7
IGHG1	53	47.5	0.52	0.176	0.52	CTD	8
H33	52.5	81	0.51	0.387	0.51	CTD	9
H31	48	60	0.42	0.255	0.42	CTD	10
AK1BA	46.5	38	0.39	0.116	0.39	CTD	12
CH60	44.5	33	0.35	0.085	0.35	CTD	13
PPIA	32	61.5	0.1	0.264	0.264	NTD	14
LDHB	38	30	0.22	0.066	0.22	CTD	15
G6PD	35.5	53	0.17	0.211	0.211	NTD	16
AK1C3	33	51	0.12	0.198	0.198	NTD	17
G3P	27	50.5	0	0.195	0.195	NTD	18
AK1C1	31	28.5	0.08	0.057	0.08	CTD	19
CAP1	28.5	19.5	0.03	0	0.03	CTD	20

**Table 3:** Scores calculated from alignments of NTD and CTD of N protein and sequences of the detected human cross-reactive antigens performed by Super algorithm. For each protein, a combined normalized score is defined as maximal normalized CTD or NTD score.

Target	Super CTD	Super NTD	Norm Super CTD	Norm Super NTD	Combined Norm Super	Driver Domain Super	Rank Super
AK1C3	117.696	32.49	1	0.205	1	CTD	1
CALR	104.314	134.133	0.837	1	1	NTD	1
AK1C1	115.998	39.463	0.979	0.259	0.979	CTD	3
AK1BA	112.491	35.35	0.937	0.227	0.937	CTD	4
HS90A	105.089	92.783	0.847	0.676	0.847	CTD	5
HSP7C	102.505	107.593	0.815	0.792	0.815	CTD	6
IGHG1	91.387	110.141	0.68	0.812	0.812	NTD	7
KPYM	102.032	53.987	0.809	0.373	0.809	CTD	8
CH60	100.863	28.215	0.795	0.171	0.795	CTD	9
G3P	95.667	39.779	0.732	0.262	0.732	CTD	10
G6PD	91.489	44.426	0.681	0.298	0.681	CTD	11
HS90B	89.986	58.397	0.663	0.407	0.663	CTD	12
LDHB	89.738	46.558	0.66	0.315	0.66	CTD	13
H33	89.177	24.762	0.653	0.144	0.653	CTD	14
H32	88.726	6.356	0.647	0	0.647	CTD	15
H2B	86.471	19.092	0.62	0.1	0.62	CTD	16
CAP1	35.53	84.841	0	0.614	0.614	NTD	17
PPIA	83.337	66.079	0.582	0.467	0.582	CTD	18
H31	82.573	14.822	0.573	0.066	0.573	CTD	19

## Data Availability

The data presented in this study are available on request from the corresponding author due to the institutional publication policy restrictions.

## Supplementary Materials

The following supporting information can be downloaded at: https://www.mdpi.com/article/10.3390/antib15010002/s1, Table S1: Mascot scoring of identified proteins. Table S2: Align and Super alignments of NTD and CTD of N protein and sequences/structures of the detected self-antigens of human origin.
