# Supplementary material for: Antibodies Against SARS-CoV-2 Nucleocapsid Protein Possess Autoimmune Properties
Source: Antibodies (Basel). 2025 Dec 22;15(1):2. doi: 10.3390/antib15010002 (PMC12821584; doi:10.3390/antib15010002)
Supplement: Supplementary file 1 [file antibodies-15-00002-s001.zip › Table S1.pdf]

Table S1. Mascot scoring of identified proteins. The threshold value was set at 56 (if score value excessed the threshold, the identification considered reliable, $p < 0.05$).

Uniprot ID	Protein	Mw (calculated), kDa	Score	Intensity coverage, %	Sequence coverage MS, %
HS90A_HUMAN	Heat shock protein HSP 90-alpha	84.6	125	48.2	39.3
HS90B_HUMAN	Heat shock protein HSP 90-beta	83.2	159	56.1	41.7
HSP7C_HUMAN	Heat shock cognate 71 kDa protein	70.9	141	34.3	47.2
HSP71_HUMAN	Heat shock 70 kDa protein 1A/1B	70.0	211	55.6	44.3
KPYM_HUMAN	Pyruvate kinase PKM	57.9	165	44.3	47.3
CH60_HUMAN	60 kDa heat shock protein, mitochondrial	61.0	58	17.0	27.2
CAP1_HUMAN	Adenylyl cyclase-associated protein 1	51.9	74	17.1	33.5
CALR_HUMAN	Calreticulin	48.1	61	10.6	36.7
G6PD_HUMAN	Glucose-6-phosphate 1-dehydrogenase	59.2	70	17.7	28.9
AK1BA_HUMAN	Aldo-keto reductase family 1 member B10	36.0	66	30.8	55.4
AK1C1_HUMAN	Aldo-keto reductase family 1 member C1	36.8	72	7.9	42.4

AK1C3_HUMAN	Aldo-keto reductase family 1 member C3	36.8	83	9.6	51.7
G3P_HUMAN	Glyceraldehyde-3-phosphate dehydrogenase	36.0	196	56.0	68.4
CA168_HUMAN	Uncharacterized protein C1orf168	82.0	61	14.5	18.3
LDHB_HUMAN	L-lactate dehydrogenase B chain	36.6	68	12.8	31.7
H31_HUMAN / H32_HUMAN / H33_HUMAN	Histone H3.1, H3.2 or H3.3	15.4	88	46.2	48.5
PPIA_HUMAN	Peptidyl-prolyl cis-trans isomerase A	18.0	110	35.1	69.7
H2B1C_HUMAN	Histone H2B type 1-C/E/F/G/I	13.9	78	15.4	63.5
IGHG1_HUMAN	Ig gamma-1 chain C region	36.1	68	50.5	40.6
