# Supplementary material for: Antibodies Against SARS-CoV-2 Nucleocapsid Protein Possess Autoimmune Properties
Source: Antibodies (Basel). 2025 Dec 22;15(1):2. doi: 10.3390/antib15010002 (PMC12821584; doi:10.3390/antib15010002)
Supplement: Supplementary file 1 [file antibodies-15-00002-s001.zip › Table S2.pdf]

PyMOL's align command employs a multi-step algorithm for structural superposition, combining sequence-based and structure-based approaches with iterative refinement. The first step involves a global dynamic programming sequence alignment of the input atom selections. This typically uses BLOSUM62 weightings, similar to BLAST. This sequence alignment establishes an initial per-residue correspondence between the two structures. Next, based on the sequence alignment, a per-atom correspondence is established. If side-chain atoms are included in the selection, they are also considered in this step. An initial structural superposition is performed further using the established atom correspondences. The Iterative Refinement (Outlier Rejection) algorithm then enters an iterative refinement phase, typically up to five cycles, to improve the fit and remove outliers. In each cycle, atoms with per-atom deviations exceeding a certain threshold (default: two standard deviations from the mean deviation) are identified as outliers. These outliers are excluded from the alignment, and the structural fit is recalculated based on the remaining atoms. This process helps to reduce the influence of structurally divergent regions (e.g., flexible loops) on the overall alignment. Finally, the number of atoms remaining after the refinement and the resulting Root Mean Square Deviation (RMSD) for those atoms are reported.

The align command is particularly effective for proteins with moderate to high sequence similarity (e.g., >30% identity). For proteins with very low sequence identity, other PyMOL commands like super (which also performs iterative refinement but without the initial sequence alignment) or cealign (a structure-based alignment algorithm) might be more suitable. The cycles and cutoff parameters of the align command allow users to control the stringency of the outlier rejection process. Increasing cycles or decreasing cutoff will lead to more aggressive outlier removal and potentially a lower RMSD, but also a smaller number of aligned atoms.

Sequence-Independent Dynamic Programming Alignment: Unlike the align command which relies on sequence alignment, super (Sequence-Independent Dynamic Programming Alignment) command in PyMOL performs a dynamic programming alignment based on structural features, independent of sequence information. This allows it to identify structurally equivalent regions even when sequence identity is low. Following the initial alignment, super executes a series of iterative refinement cycles. These cycles aim to improve the structural fit by superposition (calculating the optimal rotation and translation matrix to minimize the Root Mean Square Deviation (RMSD) between the aligned atoms), outlier rejection (identifying and removing residues or atoms that exhibit high relative variability after superposition to ensure that only the well-conserved structural core contributes to the final superposition) and re-calculation (re-evaluating the alignment and superposition based on the refined set of atoms).

Super is particularly robust for aligning proteins that share low sequence identity but maintain similar overall folds, where sequence-based methods like align might struggle. The iterative refinement process effectively focuses the superposition on the structurally conserved core regions, minimizing the influence of flexible or divergent parts. The use of dynamic programming for the initial alignment allows for flexible matching of structural elements without strict reliance on sequence order. In essence, the super algorithm in PyMOL combines a sequence-independent structural alignment with an iterative refinement process to achieve robust and accurate superpositions, especially for challenging cases involving low sequence homology.

Table S2. Align and Super alignments of NTD and CTD of N protein and sequences/structures of the detected antigens of human origin.

Target	Alignment type	Alignment illustration	Structural illustration
	Align CTD (chains A-A)	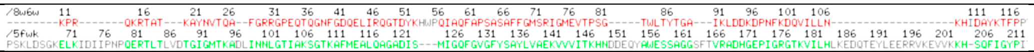	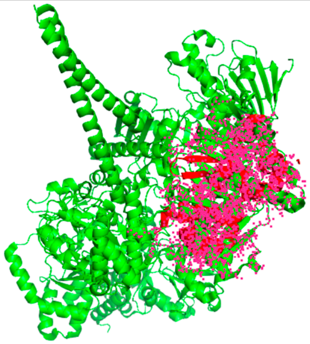
	Align CTD (chains B-A)	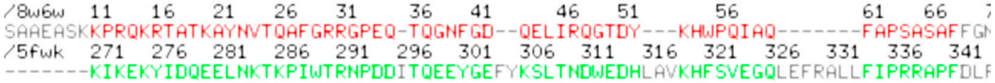	
HS90B	Super CTD (chains A-B)	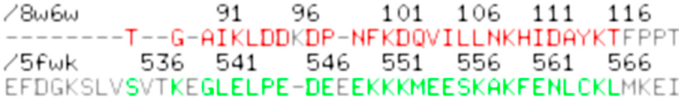	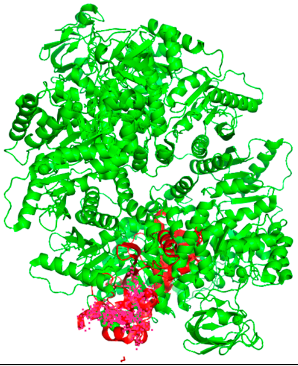
	Align NTD (chains A-D)	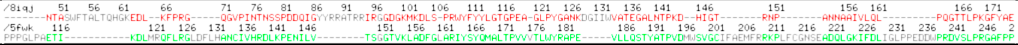	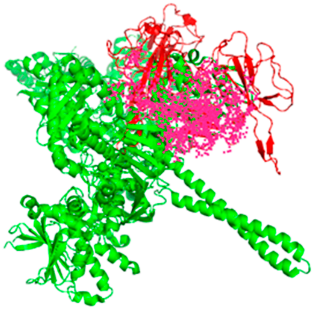
	Align NTD (chains B-D)	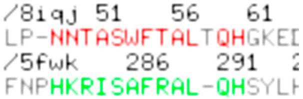	

	Super NTD (chains C-B)	/8iqj 96 101 106 111<br>---RRATRRIRGGDGKMKDLSPRWYFYYL[illegible]<br>/5fwk 156 161 166 171 17[illegible]<br>NDDEQYAWESS-AGGSFTVRADHGEPIGR[illegible]	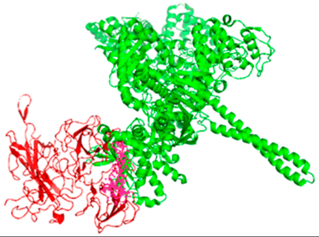
	Align CTD (chains A-A)	/8w6w 11 16 21 26 31 36 41 46 51 56 61 66 71 76 81 86 91 96 101 106 111 116<br>--------KPR--------QKRTAT---KAYNVTQA--FGRRGPEQTQGNFGDQELIRQGTDYKHWPQIAQFAPSASAFFGMSRIGMEVTPSG-----TWLTYTGA---IKLDDKDPNFKDQVILLN------------------KHIDAYKTFPP<br>/5fwk 71 76 81 86 91 96 101 106 111 116 121 126 131 136 141 146 151 156 161 166 171 176 181 186 191 196 201 206 211<br>PSKLDSGKELKIDIIPNPQERTLTLVDTGIGMTKADLINNLGTIAKSGTKAFMEALQAGADIS---MIGQFGVGFYSAYLVAEKVVVITKHNDDEQYAWESSAGGSFTVRADHGEPIGRGTKVILHLKEDQTEYLEERRVKEVVKKH-SQFIGYPI	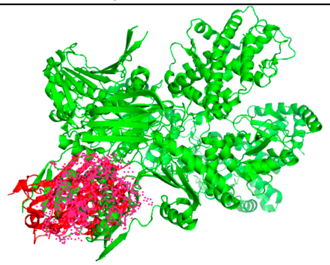
	Align CTD (chains B-A)	/8w6w 11 16 21 26 31 36 41 46 51 56 61 66 [illegible]<br>SAAEASKKPRQKRTATKAYNVTQAFGRRGPEQ-TQGNFGD--QELIRQGTDY---KHWPQIAQ-------FAPSASAFFG[illegible]<br>/5fwk 271 276 281 286 291 296 301 306 311 316 321 326 331 336 341<br>-------KIKEKYIDQEELNKTKPIWTRNPDDITQEEYGEFYKSLTNDWEDHLAVKHFSVEGQLEFRALLFIPRRAPFDL[illegible]	
HS90A	Super CTD (chains A-B)	/8w6w 91 96 101 106 111 116<br>--------T--G-AIKLDDKDP-NFKDQVILLNKHIDAYKTFPPT<br>/5fwk 536 541 546 551 556 561 566<br>EFDGKSLVSVTKEGLELPE-DEEEKKKMEESKAKFENLCKLMKEI	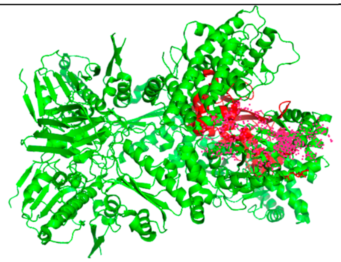
	Align NTD (chains A-A)	/8iqj 111 116 121 126 131 136 141 146 151 156 161 166<br>LSPRWYFYYLGTGPEAGLPYGANK-DGIIWVATEGALNTPKD---HIGTRNPANNAAIVLQLPQGTTLPK------<br>/7krj 71 76 81 86 91 96 101 106 111 116 121 126 131 1<br>---------LDSGKELHINLIPNKQDRTLTIVDTGIGMTKADLINNLGTIAKSGTKAFMEALQAGADISMIGQFGV	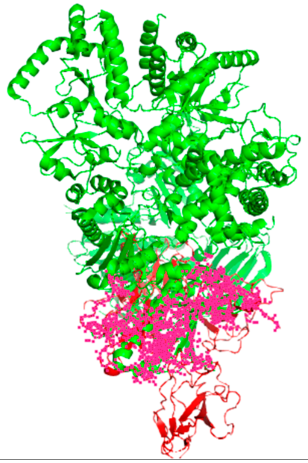
	Align NTD (chains B-A)	/8iqj 46 51 56 61 66 71 76 81 86 91 96 1<br>GSHMRPQGLPNNTASW-------FTALTQHGKEDLKFPRGQGVPINTNSSPDDQIGYYRRATRRIRGGDG[illegible]<br>/7krj 161 166 171 176 181 186 191 196 201 206<br>----------DEQYAWESSAGGSFTVRTDTGE-----PMGRGTKVILHLK-EDQTEYLEE--RRIKEIVK[illegible]	

HSP7C

Super NTD (chains D-C)

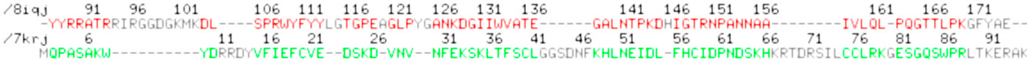

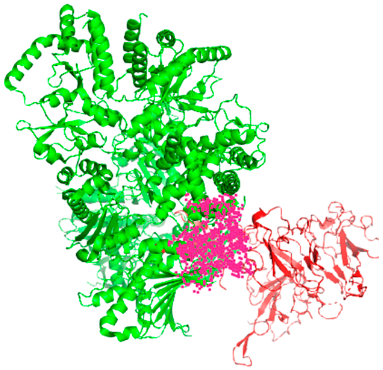

Align CTD (chains B-B)

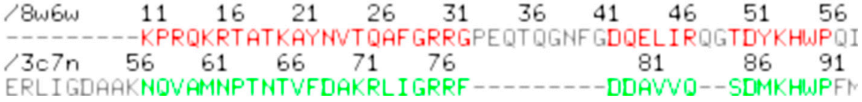

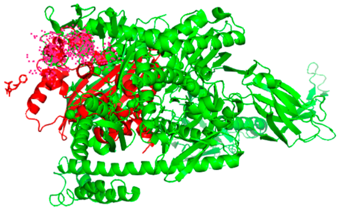

Super CTD (chains A-B)

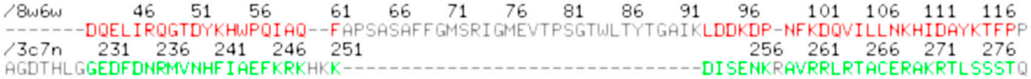

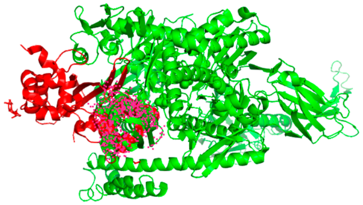

Align NTD (chains A-B)

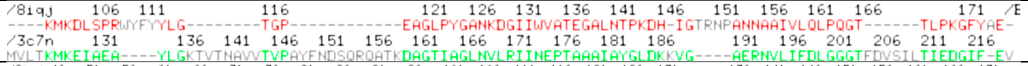

Align NTD (chains B-B)

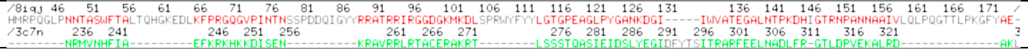

Align NTD (chains C-B)

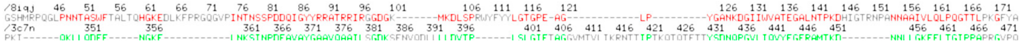

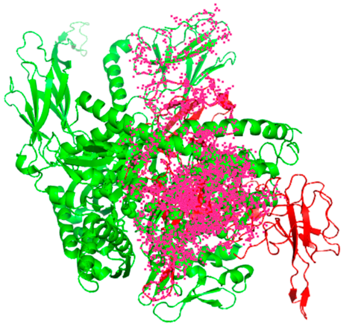

	Super NTD (chains B-A)	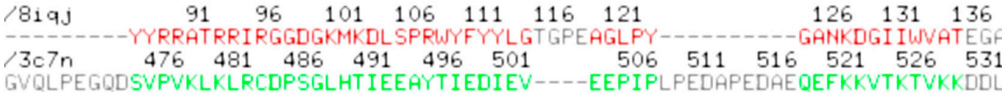	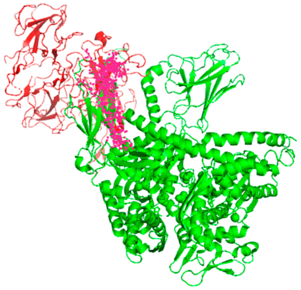
	Align CTD (chains B-A)	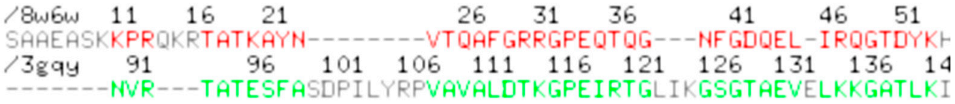	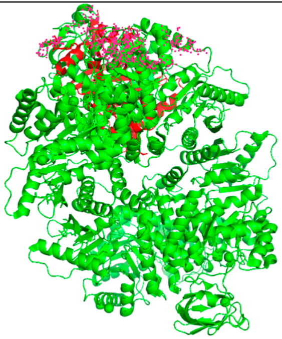
KPYM	Super CTD (chains A-D)	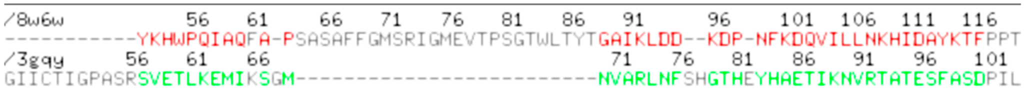	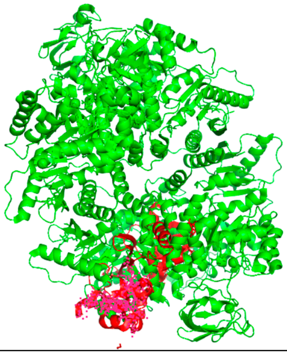
	Align NTD (chains A-A)	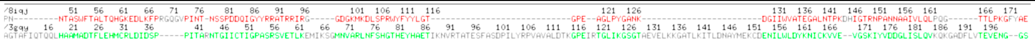	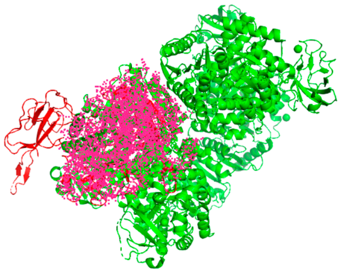
	Align NTD (chains B-A)	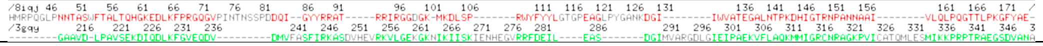	
	Align NTD (chains C-A)	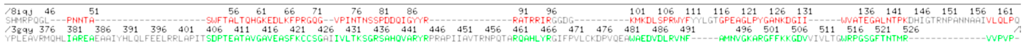	

	Super NTD (chains D-D)	/8iqj 51 56 61 66 71 76 81 86 91 96 101 106 111<br>-------NTASWFTALTQHGK-ED-LKFPRGQGVPINTNSSPDDQIGYYRRATRRIRGGDGKMKDLSP----RWYFYYL<br>/3gqy 136 141 146 151 156 161 166 171 176 181 186 191 196<br>GSGTAEVELKKGATLKITLDNAYMEKCD---ENILWLD-YKNICKVVEVGSKIYVDDGLI-SLQVKQKGADFLVTEVEN	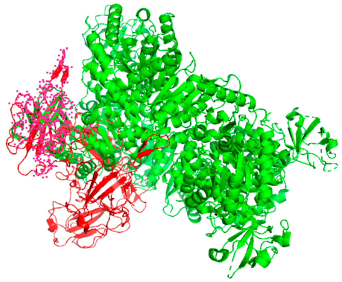
	Align CTD (chains A-A)	/8w6w 61 66 71 76 81 86 91 96 101 106<br>--------IAQFAPSASAFFGMSRIG----MEVTPSGTWLTYTG-AIKLDDKDPN----------FKDQVILLNK<br>/8g7j 271 276 281 286 291 296 301 306 311 316 321 326 331<br>RLKVGLQVVAVKAPG----FGDNRKNQLKDMAIATGGAVFGEEGLTLNLEDVQPHDLGKVGEVIVTKDDAMLLKG	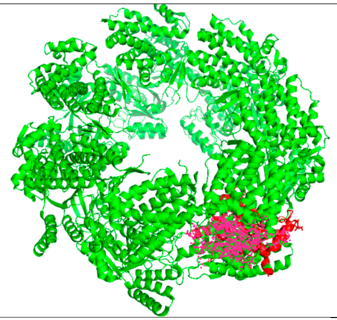
	Align CTD (chains B-A)	/8w6w 11 16 21 26 31<br>AAEASKKPRQKRTATKAYNVTQAFGRRGF<br>/8g7j 391 396 401 406<br>-------K---KDRVTDALNATRAAVEEGI	
CH60	Super CTD (chains A-F)	/8w6w 46 51 56 61 66 71 76 81 86 91 96 101 106 111 116<br>-------DQELIRQGTDYKHWPQIAQFAPSASAFFGM--SRI--GMEVTPSGTWLTYTGAIKLDDKDPNFKDQVILLNKHIDAYKTFP<br>/8g7j 336 341 346 351 356 361 366 371 376 381 386 391 396 401<br>MLLKGKGDKAQIEKRIQ--EIIEQLDVTTSEYEKEKLNERLAKLS----------DGVAVLKVGGTSDVEVNEKKDRVTDALNATRAA	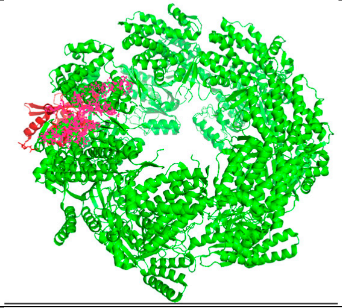
	Align NTD (chains A-A)	/8iqj 66 71 76 81<br>----EDLKFPRGQGVPINTNSSPDD<br>/8g7j 191 196 201 206<br>LEIIEGMKFDRGYISPYFINTSKGQ	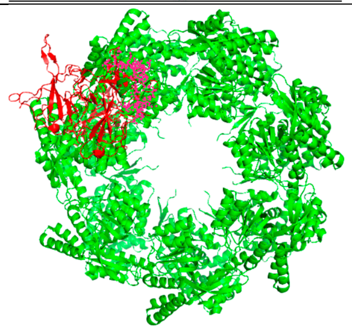

CAP1	Super NTD (chains C-A)	/8iqj 106 111 1[illegible]<br>DGKMKDLSPRWYFYYLGT[illegible]<br>/8g7j 246 251<br>--------PLVIIAEDVD[illegible]	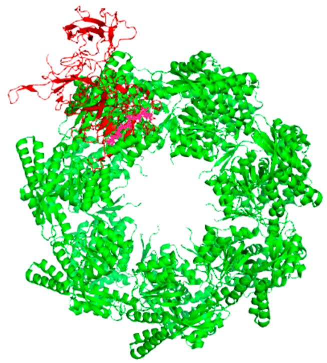
	Align CTD (chains A-A)	/8w6w 51 56 61 66 71 76 81 86<br>----------QGTDYKHWPQIAQFAPSASAFFGMSRIGMEVTPSGTWLTY<br>/1k8f 441 446 451 456 461 466 471 /B<br>SSEMNVLIPTEGGDFNEFPVPEQFKTLWN--------GQKLVTTVTEIAG	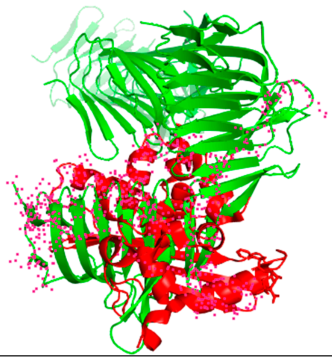
	Align CTD (chains A-B)	/8w6w 91 96 101 106 111<br>TWLTY TGAIKLDDKDPNFKDQVILLNKHIDA<br>/1k8f 319 326 331 336 341<br>TEIAG PAVLELEGKKWRVENQENVSNLVIED	
	Super CTD (chains A-A)	/8w6w 76 81 86 91<br>---------MEVTPS-GTWLTYTGAIKL<br>/1k8f 416 421 426 431 4<br>SINKTDGCHAYLSKNSLDCEIVSAKSSE	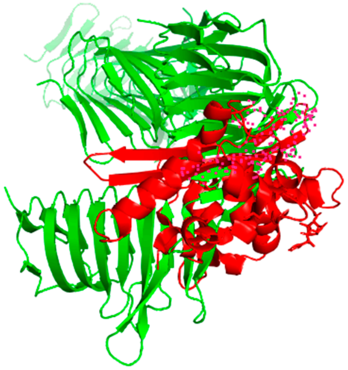

	Align NTD (chains A-A)	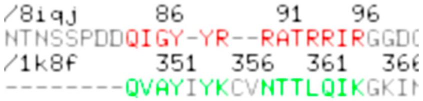	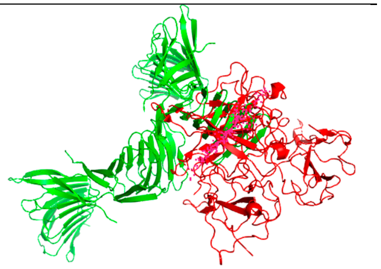
	Super NTD (chains D-D)	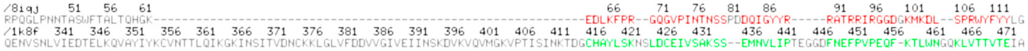	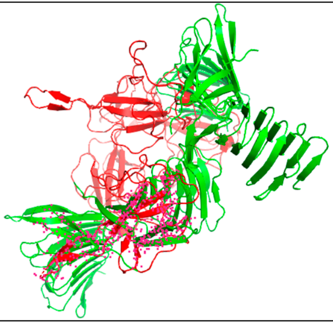
	Align CTD (chains A-C)	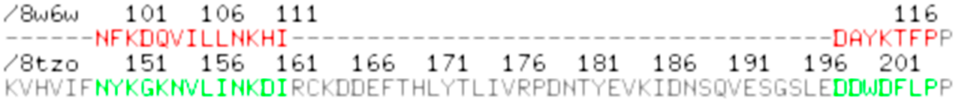	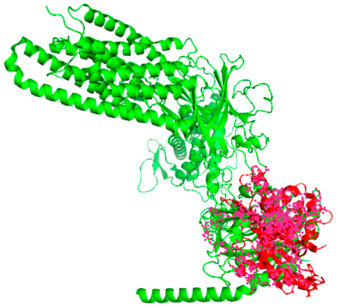
	Align CTD (chains B-C)	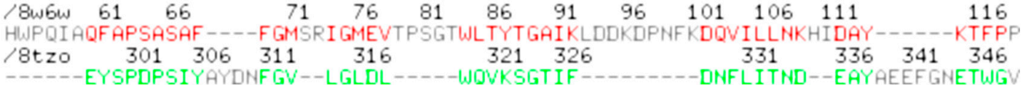	
CALR	Super CTD (chains B-B)	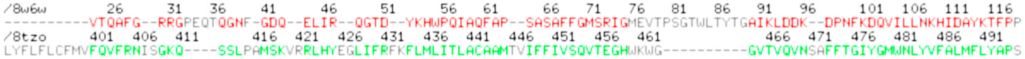	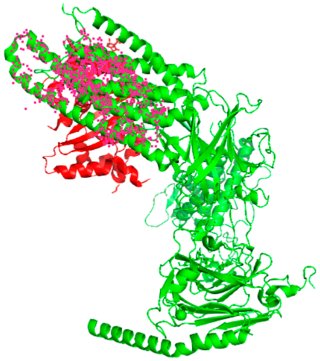

	Align NTD (chains A-B)	/8iqj 161 166 171 /E<br>---------LQLPQGTTLPKGFYAE-<br>/8tzo 36 41 46<br>IIAFLVGGLIAPGPTTAVSYMSVKC	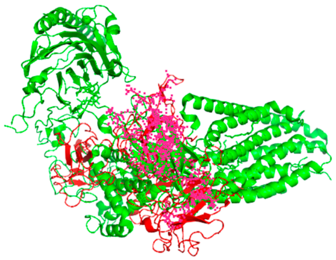
	Align NTD (chains B-B)	/8iqj 51 56 61 66 71 76 81 86 91 96<br>-------NNT---------------ASWFTALTQHGKEDLKFPRGQGVPINTNSSPDDQIGY-------------YRRATRRIRGG<br>/8tzo 86 91 96 101 106 111 116 121 126 131 136 141 146 151 156 161<br>AIPREIEANDIVFSVHIPLPHMEMSPWFQFMLFILQLDIAFKLNNQIRENAEVSMDVSLAYRDDAFAEWTEMAHERVPRKLKCT	
	Super NTD (chains D-B)	/8iqj 61 66 71 76 81 86 91 96 101 106 111 116 121 126 131 136 141 146 151 156 161<br>FTALTQHGKEDLKFPRGQ-GVPINTNSSPDDQIGYYRRATRR----IRGGDGKMKDLSPRWYFYYLGTGPEAGLPYGANKDGIIWVATEGALNTPKDHIGT--RNPANNAAIVLQLPQG<br>/8tzo 91 96 101 106 111 116 121 126 131 136 141 146 151 156 161 166 171 176 181 186<br>--------DIVFSVHIPLPHMEMSPWFQ--FMLFILQLDIAFKLNNQIREN-AEVSMDVSLAYRD--DAFA-EWTEMAHE--RVPRKLKCTFTSPKTPEHEGRYYE---CDVLPFMEIG	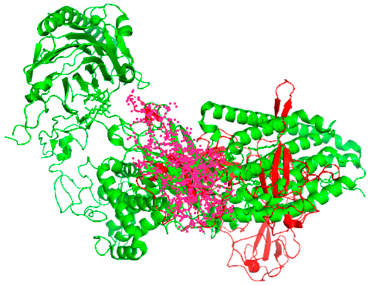
G6PD	Align CTD (chains B-A)	/8w6w 21 26 31 36 41 46 51 56 61 66 71 76 81 86 91 96 101 106 111 116<br>-------TAT-----------------------KAYNVTQAFGRRGPEQTQGNFGDQELIRQGTDYKHWPQIAQFAPSASAFFGM--SRIGMEVTP--SGTWLTYTGAIKLDDKDPNFKDQVIL-----LNKHI-------DAYKTFPP<br>/7snf 336 341 346 351 356 361 366 371 376 381 386 391 396 401 406 411 416 421 426 431 436 441 446 451 456 461 46<br>TVPRGSTTATFAAVVLYVENERWDGVPFILRCGKALNERKAEVRLQFHDVAGDIFHQQCKRNEL-------VIRVQPNEAVYTKMMTKKPGMFFNPEESELDLTYGNRYKNVK-LPDAYERLILDVFCGSQMHFVRSDELREAWRIFTP	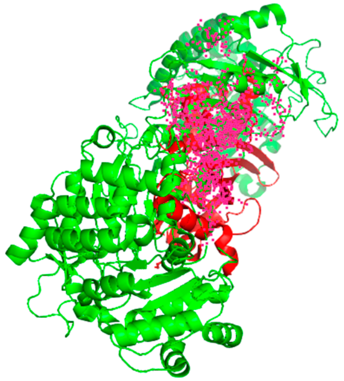

Super CTD (chains A-B)	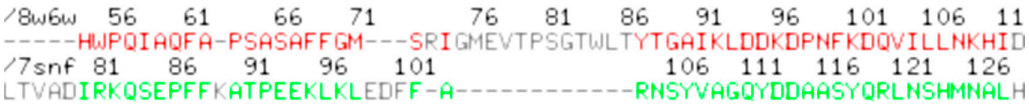	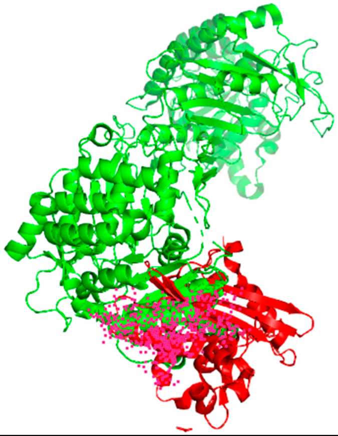
Align NTD (chains A-B)	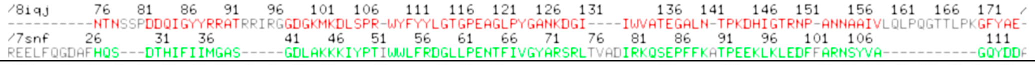	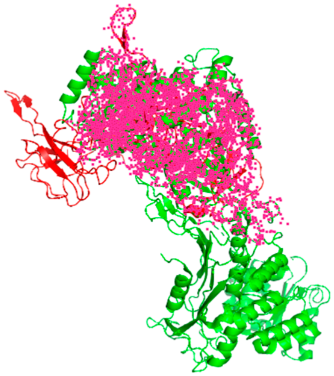
Align NTD (chains B-B)	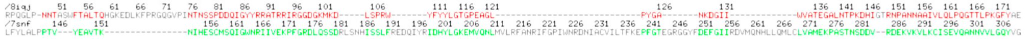	
Align NTD (chains C-B)	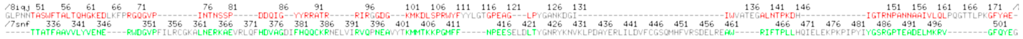	
Super NTD (chains D-A)	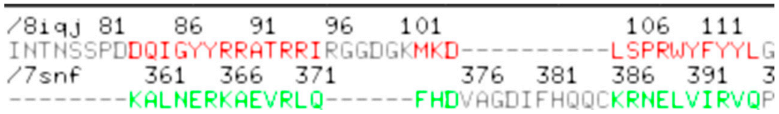	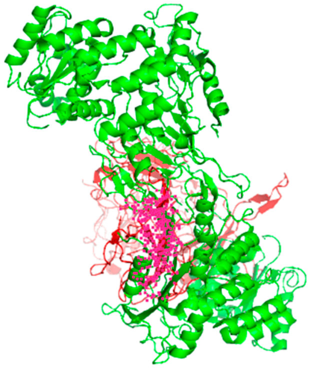

AK1B A	Align CTD (chains A-A)	/8w6w 71 76 81 86 91 96 101 106 111 116<br>ASAFFGMSRIGMEVTPSGTWLTYTGAIKLDDK---DPNFKDQVILLNKHIDAYKTFPPT<br>/4ga8 11 16 21 26 31 36 41 46 51<br>-------TKAKMPIVGLGTWKSPLGKVKEAVKVAIDAGYR--------HIDCAYVYQNE	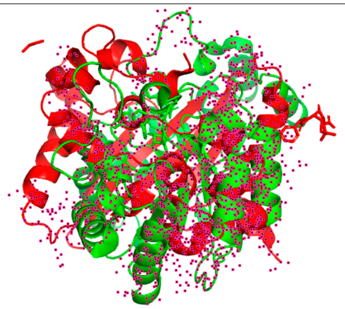
	Align CTD (chains B-A)	/8w6w 16 21 26 31 36 41 46 51 56 61 66 71 76 81 86 91 96 101 106 111 116<br>---------KRTATKAYNVTQAFGRRGPEQTQG--NFG----DQELIRQGTDYK--------H----WPQIAQFAPSASAFFGMSRIGMEVT-------PSGTWLTYTGAIKLDD----KDPNFKDQV---------ILLNKHIDAYKTFPPT<br>/4ga8 136 141 146 151 156 161 166 171 176 181 186 191 196 201 206 211 216 221 226 231 236 241 246 251 256 261<br>DDKGNAIGGKATFLDAWEAMEELVDEGLVKALGVSNFSHFQIEKLLNKPGLKYKPVTNQVECHPYLTQEKLIQYCHSK---------GITVTAYSPLGSPDRPWA------KPEDPSLLEDPKIKEIAAKHKKTAAQVLIRFHIQRNVIVIPK	
	Super CTD (chains B-A)	/8w6w /B/B/-1 6 11 16 21 26 31 36 41 46 51 56 61 66 71 76 81 86 91 96 101 106 111<br>SADSTQA- MGTKKSAAEASKKPRQKRTATKAYNVTQAFGRRGPEQTQGNFGDQELIRQG-TDYKHWPQIAQFA-PSA--SAFFG--MSRIGMEVTPSGTWLTYTGAIKLDDKDPNFKDQVILLNKHIDA<br>/4ga8 176 181 186 191 196 201 206 211 216 221 226 231 236 241 246 251 256 261 266 271 276<br>KLLNKPGL KYKPVTNQVECHPYLTQEKLIQYCHSKGITVTAYSPLGSPDRPWAKPEDPSLLEDPKIKEIAAKHKKTAAQVLIRFHIQR----------------NVIVIPK-SVTPARIVENIQVFDFKL	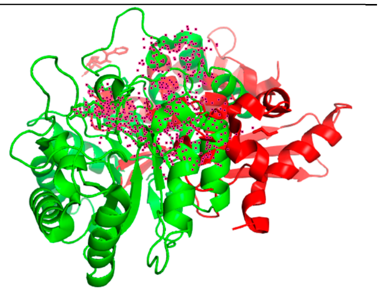
	Align NTD (chains A-A)	/8iqj 106 111 116 121 126 131 136 141 146 151 156 161 166 171 /<br>--------MKDLSPRWYFYYLGTGPEAGLPYGANKDGIIWVATEGALNTPKDHIGTRNPANNAAIVLQLPQGTT--LPKGFYAE-<br>/4ga8 96 101 106 111 116 121 126 131 136 141 146 151 15<br>VRKAFEKTLKDLKLSYLDVYLIHWPQ-GFKSG---DDLF----------PKDDKG--NAIGGKATFLDAWEAMEELVDEGLVKAL	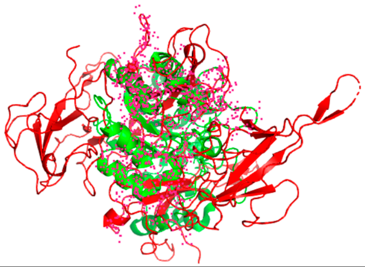
	Align NTD (chains A-B)	/8iqj 51 56 61 66 71 76<br>MRPQGLPNNTASWFTALTQHGKEDLKFPRGQGVPINTNS--<br>/4ga8 186 191 196 201 206 211<br>YKPVTN---QVECHPYLTQ--EKLIQYCHSKGITVTAYSPL	
	Super NTD (chains D-A)	/8iqj 91 96 101 106<br>QIGYYRRATRRIRGGDGKMKDLSPRW<br>/4ga8 6 11 16<br>---------FVELST----KAKMPIVG	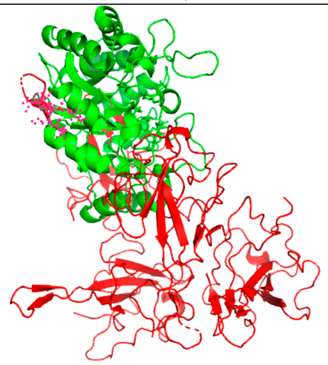

AK1C 1	Align CTD (chains A-A)	/8w6w 101 106 111 116<br>LDDKDPNFKDQVILLNK-H---IDAYKTFPP<br>/3c3u 6 11 16 21<br>--------KYQCVKLNDGHFMPVLGFGTYAP	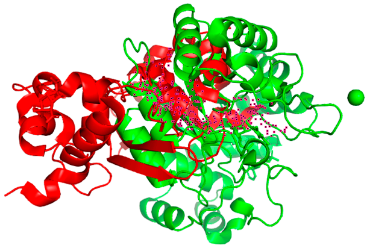
	Super CTD (chains B-A)	/8w6w 31 36 41 46 51 56 61 66 71 76 81 86 91 96 101 106 111<br>TQAFGRRGPEQTQGNFGDQELIRQ---------GTDYKHWPQIAQFA-PSA--SAFFG--MSRIGMEVTPSGTWLTYTGAIKLDDKDPNFKDQVILLNKHID<br>/3c3u 211 216 221 226 231 236 241 246 251 256 261 266 271 276 281<br>LDFCKSKDIVLVAYSALGSHREEPWVDPNSPVLLEDPVLCALAKKHKRTPALIALRYQLQR------------------GVVVLAK-SYNEQRIRQNVQVFEF	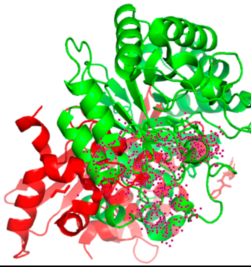
	Align NTD (chains A-A)	/8iqj 81 86 91 96 101 106<br>GVPINTNSSPDDQIGYYRRATRRIRGGDGKMKDL---SPRW<br>/3c3u 61 66 71 76 81<br>AHLYNN----EEQVGLAIRS--KIADGSVKREDIFYTSKLW	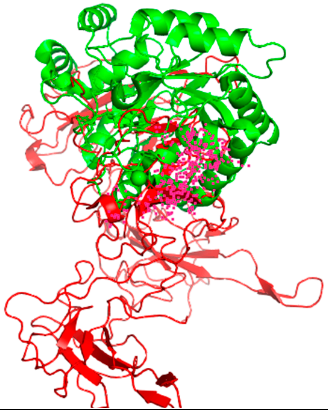
	Super NTD (chains D-A)	/8iqj 46 51 56 61<br>GSHMRPQGLPNNTASW-FTALTQHGKE<br>/3c3u 131 136 141<br>----------VIPKDENGKILFDTVDL	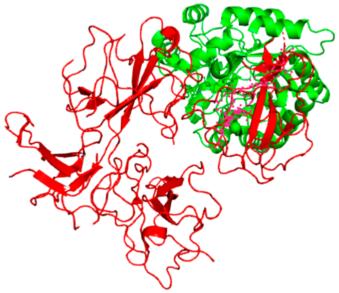

AK1C3	Align CTD (chains B-A)	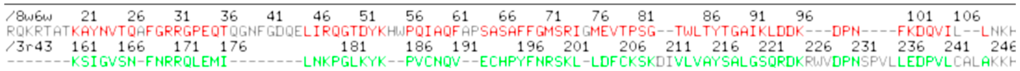	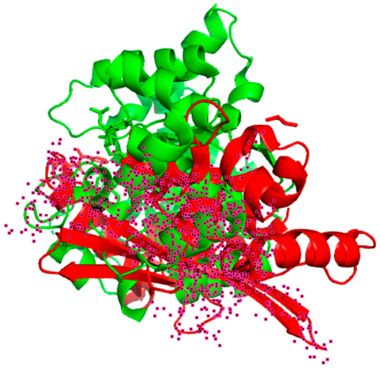
	Super CTD (chains B-A)	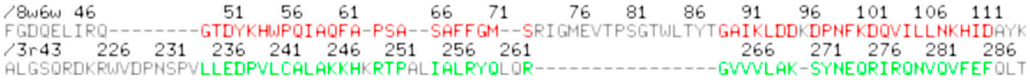	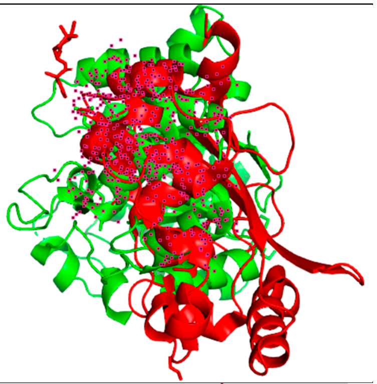
	Align NTD (chains A-A)	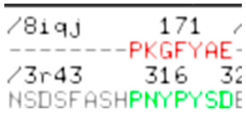	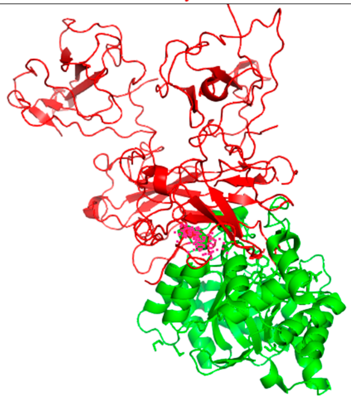

	Super NTD (chains B-A)	/8iqj 51 56 61<br>PQGLPNNTASWFTALTQHGKE<br>/3r43 11 16 2<br>---------KLNDGHFMPVLGF	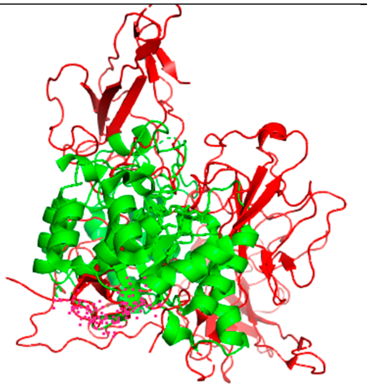
	Align CTD (chains A-B)	/8w6w 91 96 101 106 111<br>----------YTGAIKLDDKD-PNFKDQVILLNKHIDAY<br>/1u8f 306 311 316 321 326 331 /C<br>GAGIALNDHFVKLISWYDNEFGYSNRVVDLMAHMASKE-	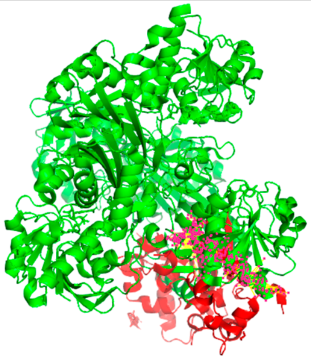
G3P	Align NTD (chains A-A)	/8iqj 71 76 81 86 91<br>--------FPRGQGVPINTN-----SSPDDQIGYYRR---------ATRR<br>/1u8f 296 301 306 311 316 321 326 331<br>SDTHSSTFDAGAGIALNDHFVKLISWYDNEFGYSNRVVDLMAHMASKE	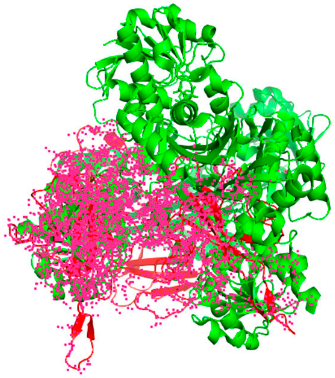
	Align NTD (chains A-B)	/8iqj 101 106 111 116 121 126 131 136 141 146 151 156 161<br>RGGDGKMKDLSPRWYFYYLGTGPEAGLPYGANKDGIIW-VATEGALNTPK-DHIGTRNPANNAAIVLQLPQG<br>/1u8f 6 11 16 21 26 31 36 41 46<br>MG---KVK---------------------VGVNGFGRIGRLVTRAAFNSGKVDIVAINDPFIDLNYMVYMFQY	
	Align NTD (chains B-B)	/8iqj 56 61 66 71 76 81 86 91 96 101 106 111 116 121 126 131 136 141 146 151 156 161 166 171<br>PNNTASWFTALTQHGKEDLKFPRGQGVPINTNSSPDDQIGYYRRATRRIRGGDGKMKDLSPRWYFYYLGTGPEAGLPYGANKDGIIWVATEGALNTPKDHIGTRNPANNAAIVLQLPQGTTLPKGFY<br>/1u8f 61 66 71 76 81 86 91 96 101 106 111 116 121 126 131<br>--------TVKAENGKLVIN-----GNPITI----------FQERDPSKIKWGD--------------------AGAEYVVESTGVF----------TTMEKAGAHLQGGAKRVIISAPSADA-PMFVM	
	Align NTD (chains C-B)	/8iqj 51 56 61 66 71 76 81 86 91 96 101 106 111 116 121 126 131 136 141 146 151 156 161 166 171<br>GLPNNTASWFTALTQHGKEDLKFPRGQGVPIN----TNSSPDDQIGYYRRATRRIRGGDGKMKDLSPRWYFYYLGTGPEAGLPYGANKDGIIWVATEGALNTPKDHIGTRN------------PANNAAIVLQLPQGTTLP-KGF--YAE-<br>/1u8f 156 161 166 171 176 181 186 191 196 201 206 211 216 221 226 231 236 241 246 251 256 261 266 271 276<br>-----TTNCLAPLAKVIHDNFGIVEGLMTTVHAITATQKTVDGPSG------KLWRDGRGALQNIIPA------STG-------AAKAVGKVIPELNGKLTGMAFRVPTANVSVVDLTCRLEKPAKYDDIKKVVKQASEGPLKGILGYTEH	
	Align NTD (chains D-B)	/8iqj 61 66 71 76 81 86 91<br>TALTQHGKEDLKFPRGQGVPINTN-----SSPDDQIGYYRR---------ATR<br>/1u8f 296 301 306 311 316 321 326 331<br>------------TFDAGAGIALNDHFVKLISWYDNEFGYSNRVVDLMAHMASK	
	Align NTD (chains D-C)	/8iqj 101 106 111 116 121 126 131 136 141 146 151<br>RGGDGKMKDLSPRWYFYYLGTGPEAGLPYGANKDGIIW-VATEGALNTPK-DHIGTRNPAN<br>/1u8f 6 11 16 21 26 31 36<br>MG---KVK---------------------VGVNGFGRIGRLVTRAAFNSGKVDIVAINDPFI	

	Super NTD (chains B-C)	/8iqj 86 91 9<br>-------GYYRRATRRIR<br>/1u8f 241 246<br>FRVPTANVSVVDLTCRLE	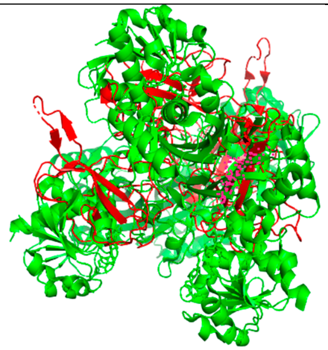
	Align CTD (chains A-A)	/8w6w 11 16 21 26 31 36 41 46 51 56 61 66 71 76 81 86 91 96 101 106 111 116 121<br>---------KPRQKRTATKAYNVTQAFGRRGPEQTQGNFGDQELIRQGTDYKHWP--------------QIAQFAPSASAFFGMSRIGMEVTPS------GTWLTYTGAIKLDDKDPNFKDQVILLNKHIDAY----KTFPPTE<br>/7dbj 226 231 236 241 246 251 256 261 266 271 276 281 286 291 296 301 306 311 316 321 326 331 /B/[illegible]<br>GTDNDSENWKEVHKMVVESAYEVIKLKG----------------------YTNWAIGLSVADLIESMLKNLSRIHPVSTMVKGMYGIENEVFLSLPCILNARGLTSVINQKLKD------DEVAQLKKSADTLWDIQKDLKDL-	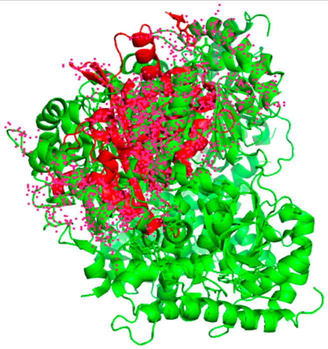
LDHB	Align CTD (chains B-B)	/8w6w 11 16 21 26 31 36 41 46 51 56 61<br>AEASK--------KPRQKRTATKAYNVTQ----AFGRRGPEQTQGNFGDQELIRQGTDYKHW--PQIAQFAPSA<br>/7dbj 71 76 81 86 91 96 101 106 111 116 121 126 13<br>EMMDLQHGSLFLQTP--KIVADKDYSVTANSKIVVVTAGVRQQEGE-SRLNLVQRNVNVFKFIIPQIVKYSPDC	
	Super CTD (chains A-A)	/8w6w 96 101 106 111 116 1<br>-------DDKDPNFKDQVILLNKHIDA-YKTFPPTE<br>/7dbj 221 226 231 236 241 246<br>FLNPEMGTDNDSENWKEVHKMVVESAYEVIKLKGY	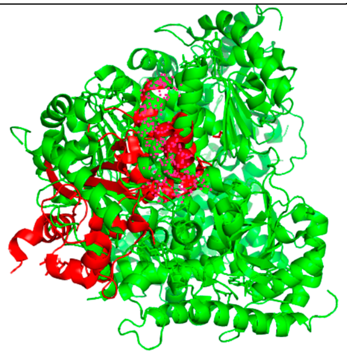

	Align NTD (chains A-A)	/8iqj 86 91 96 101 106 111 116 121 126 131 136 141 146 151 156<br>---------GYYRRATRRIRG---------GDGKMKDLSPRWYFYY---LGTGPEA--GLPYGANKDG--IIWVATEGA---LNTPKDHIGTRNPANNAAI<br>/7dbj 146 151 156 161 166 171 176 181 186 191 196 201 206 211 216 221 226 2<br>VSNPVDILTY---VTWKLSGLPKHRVIGSGCNLD-SARFRYLMAEKLGIHPSSCHGWILGEHGDSSVAVWSGVNVAGVSLQELNPEMGTDNDSENWKE	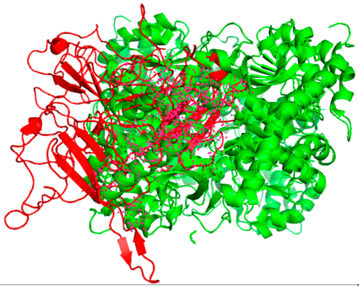
	Super NTD (chains A-A)	/8iqj 101 106 111 116<br>RIRGGDGKMKDLSPRWYFYYLGTGPE<br>/7dbj 11 16 21 2<br>--------LIAPVAEEEATVPNNKITV	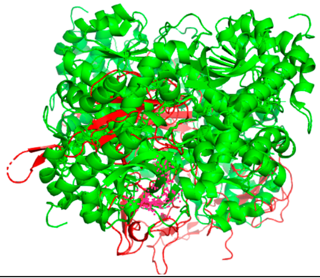
	Align CTD (chains A-D)	/8w6w 36 41 46 51 56 61 66 71 76 81 86 91 96 101 106 111 116 121 126<br>-----PEQTQGNFGDQELIRQGTDYKHWPQIAQFAPSASAFFGM----------SRIGMEVTPSGTWLTYTGAIKLDDKDPNFKDQVIL---LNKHIDAYKTFPPTEPKKDKK<br>/1u35 1236 1241 1246 1251 1256 1261 1266 1271 1276 1281 1286 1291 1296 1301 1306 1311 1316 /G<br>RKRGRKESYSIYV------------YKVLKQVHPDTGISSKAMGIMNSFVNDIFERIASEASRLAH---YNKRSTITSREVQTAVRLLLPGELAKHAVSEGTKAVTKYTSSK-	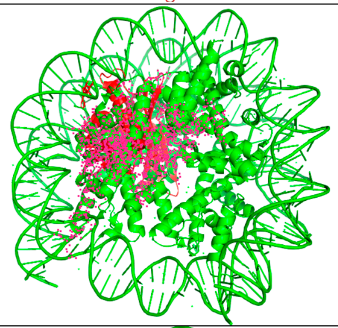
	Align CTD (chains B-E)	/8w6w 16 21 26 31 36 41 46 51 56<br>AEASKKPRQKRTATKAYNVTQAFGRRGPEQTQGNFGDQELIRQ-GTDYKHWPQ<br>/1u35 641 646 651 656 661 666 671 676 681<br>----------YRPGTVALREIRRY-QKSTELLIRKLPFQRLVREIAQDFKTDLR	
**H31**	Super CTD (chains B-G)	/8w6w 101 106 111 116<br>-----NFKDQVILLNKHIDAYKTFPP<br>/1u35 1051 1056 1061 1066<br>GVGAPVYMAAVLEYLTAEILELAVNA	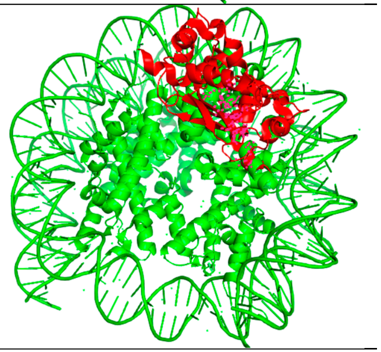

	Align NTD (chains A-B)	/8iqj 56 61 66 71 76 81 86 91 96 101 106 111 116 121 126 131 136 141 146 151 156 161 166 171<br>---------AL TQHGKEDLKFPRGQGVPINTNSSPDDQIGYYRRATRRI--RGGDGKMKDLSPRWYFYYLGTGPEAGLPYGANKDGIIWVATEGAL-----NTPKDHI-----GTRNPANNAAIVLQLP-QGTTLPKGFYAE<br>/1u35 /D/B/0 6 11 16 21 26 31 36 41 46 51 56 61 66 71 76 81 86 91 96 /E/C/<br>LARRIRGERA- MSGRGKGGKGLGKGGAKRHRKVLRDNIQGITKPAIRRLARRGG---VKRIS----------------------GLIYEETRGVLKVFLENVIRDAVTYTEHAKRKTVTAMDVVYALKRQGRTL-YGFGG-	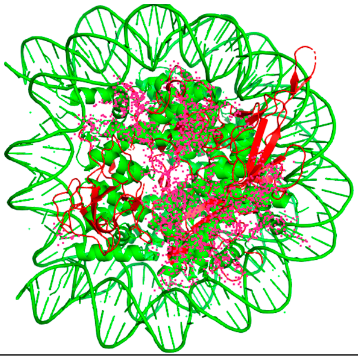
	Align NTD (chains B-C, C-C)	/8iqj 56 61 66 71 76 81 86 91 96 101 106 111 116 121 126 131 136 141 146 151 156 161 166 171 /C/C/21 26 31 36 41 46 51<br>NNTASWFTALTQHGKEDLKFPRGQGVPINTNSSPDDQIGYYRRATRRIRGGDGKMKDLSPRWYFYYLGTGPE----AGLPYGANKDGIIWVATEGALNTPKDHIGTRN----PANNAAIVLQLPQGTTLPKGFYAE- MGSSHHHHHHSSGLVPRGSHMRPQGLPNNTASW<br>/1u35 816 821 826 831 836 841 846 851 856 861 866 871 876 881 886 891 896 901 906 911 916 /F<br>--------KTSRSAKAGVIFPVG------------------RMLRYIKKGHPK----------YRIGVGAPVYMAAVLEYLTAE--ILELAVNAARDNKKGRVTPRHILLAVANDEELN-QLLKGVTIASGGVLPN IHPELL-------------------AKKRGS-	
	Super NTD (chains A-A)	/8iqj 76<br>---------PINTN<br>/1u35 436 44<br>PATGGVKKPHRYR	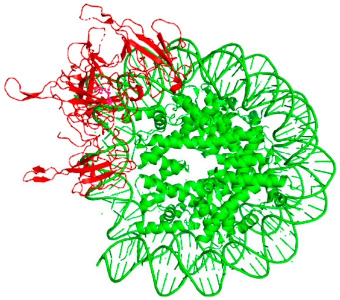
	Align CTD (chains A-A)	/8w6w 101 106<br>-----PNFKDQVILLNKH<br>/1tzy 111 116<br>QGGVLPNI--QAVLLPKK	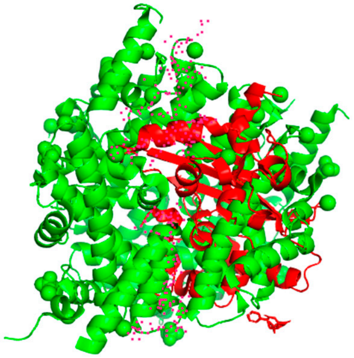
**H32**	Align CTD (chains B-B)	/8w6w 11 16<br>---------KPRQKRTATK<br>/1tzy 81 86<br>RIAGEASRLAHYNKRSTI	

Super CTD (chains A-E)	/8w6w 41 46 51 56 61 66<br>--------NFGDQELIRQGT-DYKHWPQIAQFAPSASAI<br>/1tzy 76 81 86 91 96 101<br>NAARDNKKTRIIPRHLQLAIRNDEELNKLLGKVTIAQGI	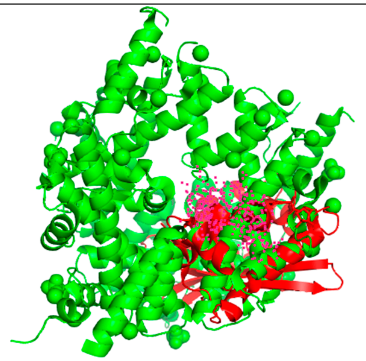
Align NTD (chains A-A)	/8iqj 161 166 171 /<br>NPANNAAIVLQLPQGTT---LPKGFYAE-<br>/1tzy 26 31 36 41<br>----------LQFPVGRVHRLLRKGNYAER	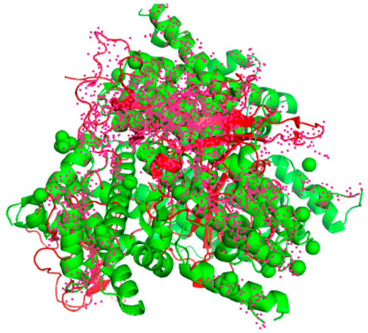
Align NTD (chains B-A)	/8iqj 56 61<br>------FTALTQHGKED<br>/1tzy 111 116 1<br>GGVLPNIQAVLLPKKTD	
Align NTD (chains C-D)	/8iqj 131 136 141 146 151 156<br>-------DGIIWVATEGAL-----NTPKDHIGTRNPANNAAIV-<br>/1tzy 46 51 56 61 66 71 76 81<br>RGGVKRISGLIYEETRGVLKVFLENVIRDAVTYTEHAKRKTVTAI	
Align NTD (chains B-B)	/8iqj 106 111 116 121 126 131 136 141 146 151 156 161 166 171<br>KMKDL--SPRWYFYYL-----GTGPEAGLPYGANKDGII---------WVATEGALNTPKDHIGTRNPANN----AAIVLQLP---------QGTTLPKGFYAE<br>/1tzy 36 41 46 51 56 61 66 71 76 81 86 91 96 101 106 111 116 121<br>DKKRKKSRKESYSIYVYKVLKQVHPDTGISSKAM--GIMNSFVNDIFERIAGEASRLA---HYNKRSTITSREIQTAVRLLLPGELAKHAVSEGTKAVTKYTSS	
Align NTD (chains C-C)	/8iqj 51 56 61 66 71 76 81 86 91 96 1(<br>----------PNNTASWFTALT----QHGKEDLKFPRGQGVPINTNSSPDDQIGYY-----------RRAT----------RRIRGGDGK<br>/1tzy 66 71 76 81 86 91 96 101 106 111 116 121 126 131 /(<br>STELLIRKLP-------FQRLVREIAQDFKTDLRF-QSSAVMALQEASEAYLVGLFEDTNLCAIHAKRVTIMPKDIQLARRIRGERA-	
Align NTD (chains C-E, D-E, D-F)	/8iqj 161 166 171 /D/D/21 26 31 36 41 46 51 56 61 66 71 V P 76 81 86 91 96 101 106 111 116 121<br>--------LQLPQGTT---LPKGFYAE- MGSSHHHHHHSSGLVPRGSHMRPQGLP-------------------------------------NNTASWFTALTQHGKEDLKFPRGQG INTNSSPDDQIGYYRRATRRIRGGDGKMKDL--SPRWYFYYL-----GTGPEAGLPY<br>/1tzy 21 26 31 36 41 46 51 56 61 66 71 76 81 86 91 96 101 106 111 116 121 /EA/G/ /F/F/0 6 11 16 21 26 31 36 41 46 51 5<br>KSRSSRAGLQFPVGRVHRLLRKGNYAER VGAGAPVYLAAVLEYLTAEILELAGNAARDNKKTRIIPRHLQLAIRNDEELNKLLGKVTIAQGGVLPN-----IQAVLLPKKTDSHKAKAK- CL - MPEPAKSAPAPKKGSKKAVTKTQKKGDKKRKKSRKESYSIYVYKVLKQVHPDTGISS	
Super NTD (chains B-D)	/8iqj 81<br>---------SSPDD<br>/1tzy 21<br>GGAKRHRKVLRDN	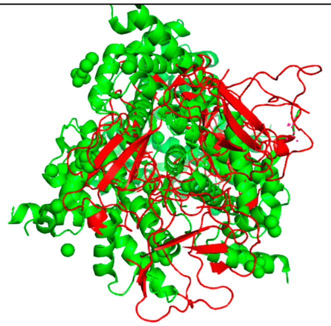

<table>
<tr><td rowspan="6">H33</td><td>Align CTD (chains A-C)</td><td>/3wtp 111 116 121
GGVLPNI--QAVLLPKKTESHHK
/8w6w 101 106 111 1
----PNFKDQVILLNKHIDAYKT</td><td rowspan="2">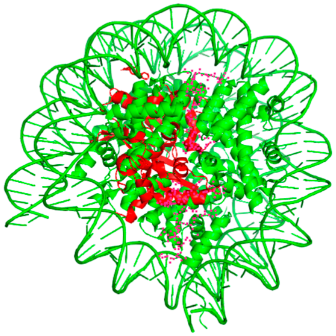</td></tr>
<tr><td>Align CTD (chains B-D)</td><td>/3wtp 81 86
ERIAGEASRLAHYNKRSTI
/8w6w 11 16
----------KPRQKRTATK</td></tr>
<tr><td>Super CTD (chains A-C)</td><td>/3wtp 76 81 86 91 96 101
AGNAARDNKKTRIIPRHLQLAIRNDEELNKLLGRVTIAQ[illegible]
/8w6w 41 46 51 56 61 6[illegible]
----------NFGDQELIRQGT-DYKHWPQIAQFAPSAS[illegible]</td><td>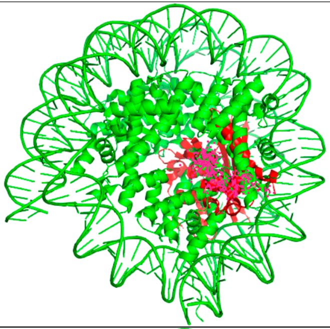</td></tr>
<tr><td>Align NTD (chains A-E)</td><td>/8iqj 141 146 151 156 161 166 171 /[illegible]
---------LNTPKDH--IGTRNPANNAAI----VLQLPQGTTLPKGFYAE-
/3wtp 91 96 101 106 111 116 121 126 131
FQSAAIGALQEASEAYLVGLFEDTNLCAIHAKRVTIMPKDIQLARRIRGER</td><td rowspan="3">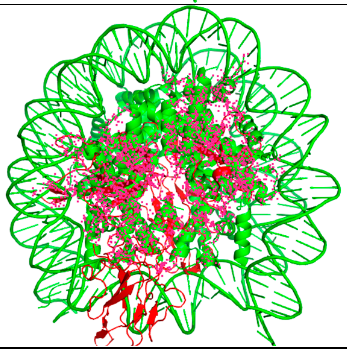</td></tr>
<tr><td>Align NTD (chains B-F)</td><td>/8iqj 66 71 76 81 86 91 96 101 106 111 116 121 126 131 136 141 146 151 156 161 166 171 /
TQHGKEDLKFPRGQGVPINTNSSPDDQIGYYRRATRRI--RGGDGKMKDLSPRWYFYYLGTGPEAGLPYGANKDGIIWVATEGAL-----NTPKDHI-----GTRNPANNAAIVLQLP-QGTTLPKGFYAE-
/3wtp 21 26 31 36 41 46 51 56 61 66 71 76 81 86 91 96 1
------HRKVLR------------DNIQGITKPAIRRLARRGG---VKRIS----------------------GLIYEETRGVLKVFLENVIRDAVTYTEHAKRKTVTAMDVVYALKRQGRTL----YGFG</td></tr>
<tr><td>Align NTD (chains C-G)</td><td>/8iqj 56 61 66 71 76 81 86 91 96 101 106 111 116 121 126 131 136 141 146 151 156 161 166 171 /
NTASWFTALTQHGKEDLKFPRGQGVPINTNSSPDDQIGYYRRATRRIRGGDGKMKDLSPRWYFYYLGTGPEAGLPYGANKDGIIWVATEGALNTPKDHIGTRNPANNAAIVLQ-------------LPQGTTLPKGFYAE-
/3wtp 16 21 26 31 36 41 46 51 56 61 66 71 76 81 86 91 96 101 106 111 1
-------AKTRSSRAGLQFPVG------------------RVHRLLRKGN----------YSERVGAGAPVYL--AAVLEYLTAEILELAGNAARDNKKTRIIPRHLQLAIRNDEELNKLLGRVTIAQGGVLPNIQAVLL</td></tr>
</table>

	Super NTD (chains A-E)	/8iqj 161<br>----AIVLQLPQG<br>/3wtp 41<br>TGGVKKPHRYRPG	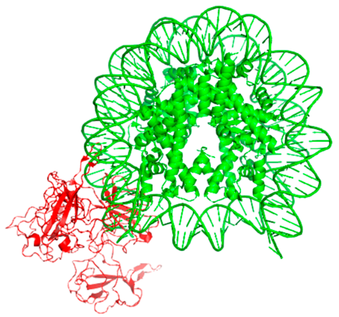
	Align CTD (chains A-C)	/8w6w 86 91 96 101 106 111<br>----------WLTYTGAIKLDDKDPNFKDQVIL-LNKHIDAYK<br>/1ak4 516 521 526 531 536 541 /<br>TTSTLQEQIGWMTHNPPIPVGEI---YKRWIILGLNKIVRMY-	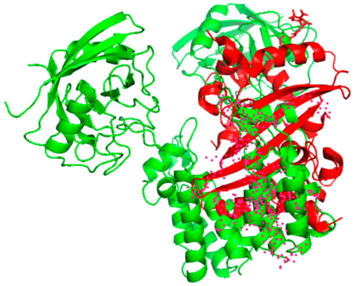
PPIA	Super CTD (chains A-C)	/8w6w 101 106 111 116 12<br>-------NFKDQVILLNKHIDAYKTFPPTEF<br>/1ak4 466 471 476 481 486<br>VGGHQAAMQMLKETINEEAAEWDRLHPVHAG	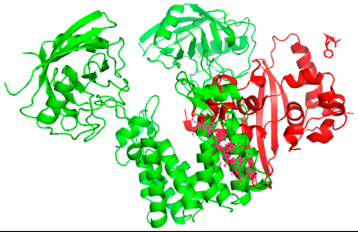
	Align NTD (chains A-A, A-B)	/8iqj 61 66 71 76 81 86 91 96 101 106 111 116 121 126 131 136 141 146 151 156 161 166 171 /<br>-------LTQH----GK-------EDLKF---PRGQGVPINTNSSPDDQIGYYRRATRRIRGGDGKMKDLSPRWYFYYLGTGPEAGLPYGANKDGIIWVATEGALNTPKDHIGTRNPANNAAIVL----QL- ---------------PQG--------------------TTLPKGFYAE-<br>/1ak4 66 71 76 81 86 91 96 101 106 111 116 121 126 131 136 141 146 151 156 161 /B/B/1 6 11 16 21 26 31 36 41 46<br>FMCQGGDFTRHNGTGGKSIYGEKFEDENFILKHTGPGILSMANAGPNTNGSQFFICTAKTEWLDGKH------------------VVFGKVKEGMNIVEA-------MERFGSRNGKTSKKITIADCGQLE MVNPTVFFDIAVDGEPLGRVSFELFADKVPKTAENFRALSTGEKGF-GYK	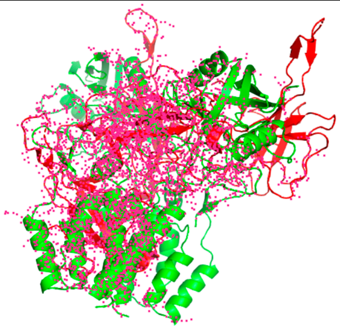
	Align NTD (chains B-B, B-C)	/8iqj 51 56 61 66 71 76 81 86 91 96 101 106 111 116 121 126 131 136 141 146 151 156 161 166 171 /<br>MRPQGLPNNTASWFTALTQHGK--EDLKF---PRGQGVPINTNSSPDDQIGYYRRATRRIRGGDGKMKDLSPRWYFYYLGTGPEAGLPYGANKDGIIWVATEGALNTPKDHIGTRNPANNAAI--------- -VLQLPQGTTL-----------------PKGFYAE-<br>/1ak4 76 81 86 91 96 101 106 111 116 121 126 131 136 141 146 151 156 161 /C/C/401 406 411 416 421 426 431 4<br>HNGTG--GKSI--------YGEKFEDENFILKHTGPGILSMANAGPNTNGSQFFICTAKTEWLDGKH------------------VVFGKVKEGMNIVEA-------MERFGSRNGKTSKKITIADCGQLE PIVQNLQGQMVHQAISPRTLNAWVKVVEEKAFSPEV	
	Align NTD (chains C-C, C-D)	/8iqj 51 56 61 66 71 76 81 86 91 96 101 106 111 116 121 126 131 136 141 146 151 156 161 166 171 /I<br>-------PNNTASWFTALTQHGKE----DLKFPRGQGVPINTNSSPDDQIGY-------------YRR---------ATRR- --IRGGDGKM--KDLSPRWYFYYLGT------GPEAGLPYGANKDGIIWVATEGALNTPKDHIGTRNPANNAAIVLQLPQGTTLPKGFYAE-<br>/1ak4 476 481 486 491 496 501 506 511 516 521 526 531 536 541 /D/D/401 406 411 416 421 426 431 436 441 446 451 456 461 466 471 476<br>QMLKETINEEAAEWDRLHPVHAGPIAPGQMREPRGSDIA-GTTSTLQEQIGWMTHNPPIPVGEIYKRWIILGLNKIVRMY PIVQNLQGQMVHQAISPRTLNAWVKVVEEKAFSPEVIPMFSALSEG----ATPQDLNTMLNTVGGHQAA------MQMLKETINEEA--AEW	
	Align NTD (chains D-D)	66 71 76 81 86<br>DLKFPRGQGVPINTNSSPDDQIGYYR<br>496 501 506 511 516<br>--MREPRGSDIA-GTTSTLQEQIGWMT	

	Super NTD (chains C-B)	/8iqj 86 91 96 101 106 111 1<br>NTNSSPDDQIGYYRRATRRIRGGDGKMKDLSPRWYFYYLGT<br>/1ak4 6 11 16 21 26<br>-----------NPTVFFDIAV----DGEPLGRVSFELFADK	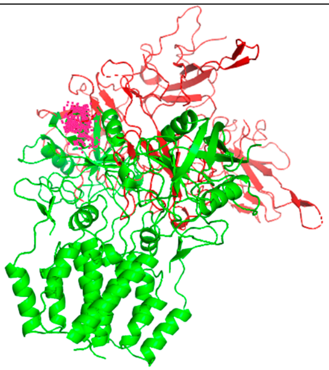
	Align CTD (chains A-C)	/8w6w 101 106 111<br>---PNFKDQVILLNKHIDAYK<br>/3x1s 116 121<br>GVLPNI--QAVLLPKKTESHK	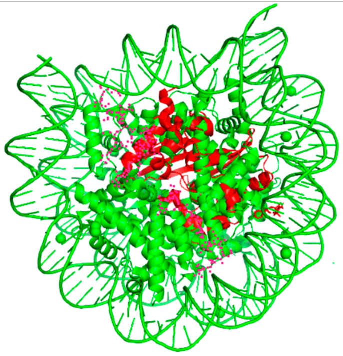
	Align CTD (chains B-D)	/8w6w 11 16<br>--------KPRQKRTATK<br>/3x1s 81 86<br>RIAGEASRLAHYNKRSTI	
**H2B**	Super CTD (chains B-C)	/8w6w 41 46 51 56 61 66<br>--------NFGDQELIR-QGTDYKHWPQIAQFAPSASAF<br>/3x1s 76 81 86 91 96 101 1<br>NAARDNKKTRIIPRHLQLAIRNDEELNKLLGRVTIAQGG	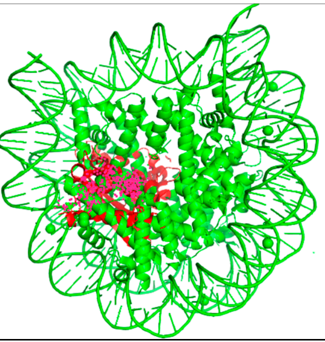

	Align NTD (chains A-B)	/8iqj 86 91 96 101 106 111 116 121 126 131 136 141 146 151 156 161 166 171<br>-------DQIGYYRRATRRI--RGGDGKMKDLSPRWYFYYLGTGPEAGLPYGANKDGIIWVATEGAL-----NTPKDHI-----GTRNPANNAAIVLQLP-QGTTLPKGFYAE<br>/3x1s 26 31 36 41 46 51 56 61 66 71 76 81 86 91 96 /C<br>HRKVLRDNIQGITKPAIRRLARRGG---VKRIS----------------------GLIYEETRGVLKVFLENVIRDAVTYTEHAKRKTVTAMDVVYALKRQGRTL-YGFGG-	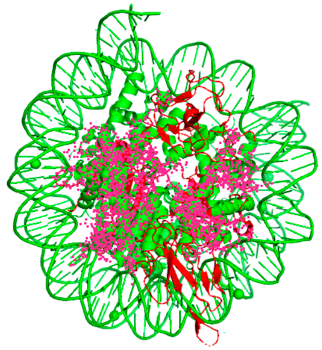
	Align NTD (chains B-C)	/8iqj 56 61 66 71 76 81 86 91 96 101 106 111 116 121 126 131 136 141 146 151 156 161 166 171 /<br>TASWFTALTQHGKEDLKFPRGQGVPINTNSSPDDQIGYYRRATRRIRGGDGKMKDLSPRWYFYYLGTGPEAGLPYGANKDGIIWVATEGALNTPKDHIGTRNPANNAAIVLQ-------------LPQGTTLPKGFYAE-<br>/3x1s 16 21 26 31 36 41 46 51 56 61 66 71 76 81 86 91 96 101 106 111 1<br>------AKTRSSRAGLQFPVG-------------------RVHRLLRKGN----------YSERVGAGAPVYL--AAVLEYLTAEILELAGNAARDNKKTRIIPRHLQLAIRNDEELNKLLGRVTIAQGGVLPNIQAVLL	
	Super NTD (chains D-D)	/8iqj 61 66<br>TALTQHGKEDLKFPRG<br>/3x1s 31 36<br>---------RSRKESY	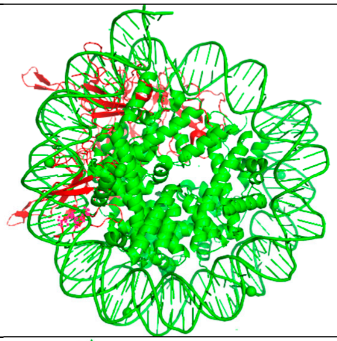
	Align CTD (chains A-E)	/8w6w 31 36 41 46 51 56 61 66 71 76 81 86 91 96 101 106 111 116 121<br>-----RGPEQTQGNFGDQELIR----QG-TDYKHW--------PQIAQFAPSA------SAFFGMSRIGMEVTPSGTWLTYT-GAIKLDDKDPNFKDQVILLNKHIDAY---------KT--------FPPTEPKI<br>/7czr 1 6 11 16 21 26 31 36 41 46 51 56 61 66 71 76 81 86 91 96 101 106 111 116 121<br>DIQLTQSPSFLSASVGDRVTITCRASQGISSYLAWYQQKPGKAPKLLIYAASTLQSGVPSRFSGSG--------SGTEFTLTISSLQPEDFATYYCQQ---LNSYPPAFGGGTKVEIKRTVAAPSVFIFPPSDEQI	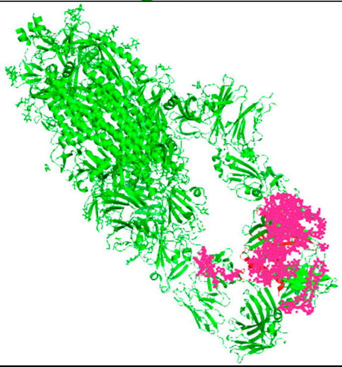
IGHG 1	Align CTD (chains B-E)	/8w6w -1 6 11 16 21 26 31 36 41 46 51 56 61 66 71<br>QA- MGTKKSAAEASKKPRQKRTATKAY--NVTQAFGRRGPEQTQGNFGD- --- --- -QELIRQG---------------------TDYKHWPQ---------IAQFAPSASAFFGMSR<br>/7czr 176 181 186 191 196 201 206 211 /EA/A/ /EB/C/ /F/J/1 6 11 16 21 26 31 36 41 46 51 56 61<br>YSL SSTLTLSK----ADYEKH---KVYACEVTHQ-GLSSPVTKSFNRGEC NAG NAG EVQLVESGGGLIQPGGSLRLSCAASGFTVSSNYMSWVRQAPGKGLEWVSVIYPGGSTFYADSV	

Super CTD (chains B-B)	/8w6w 101 106 111 116<br>-----NFKDQVILLNKHIDAYKTFPF<br>/7czr 951 956 961 966<br>LGKLQDVVNQNAQALNTLVKQLSSNF	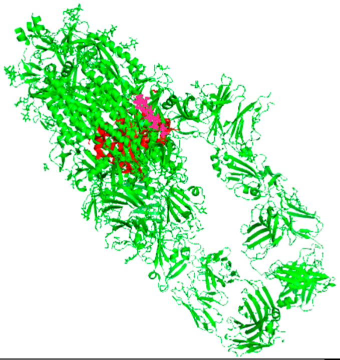
Align NTD (chains B-E)	/8iqj 76 81 86 91 96 101 106 111 116 121 126 131 136 141 146 151 156 161 166 171<br>--- PINTNSSPD---DQIGYYRRATRRIRGGDGKMKDLSPRWYFYYLGTGP----------EAGLP--YGANKDGIIWVATEGALNTPKD-------HIGTRNPANNAAIVLQLPQGTTLPKGFYA<br>/7czr /E/K/1 6 11 16 21 26 31 36 41 46 51 56 61 66 71 76 81 86 91 96 101 106 111 116<br>NAG DIQLTQSPSFLSASVG--DRVTITCRASQGISSYLA--WYQQKPGKAPKLLIYAASTLQSGVPSRFSGSGSGTEFTLTISSLQ-PEDFATYYCQQLNSYPPAFGGGTKVEIKRTVAAPSVFIF	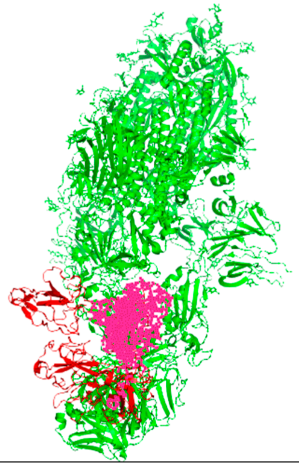

Super NTD (chains D-G)	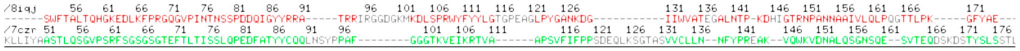	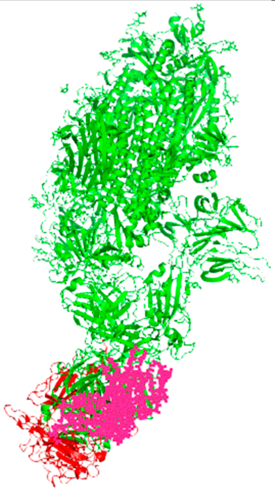
